# Most Plastic Products Release Estrogenic Chemicals: A Potential Health Problem That Can Be Solved

**DOI:** 10.1289/ehp.1003220

**Published:** 2011-03-02

**Authors:** Chun Z. Yang, Stuart I. Yaniger, V. Craig Jordan, Daniel J. Klein, George D. Bittner

**Affiliations:** 1 CertiChem Inc., Austin, Texas, USA; 2 PlastiPure Inc., Austin, Texas, USA; 3 Lombardi Comprehensive Cancer Center, Georgetown University Medical Center, Washington, DC, USA; 4 Neurobiology Section, School of Biology, University of Texas, Austin, Texas, USA

**Keywords:** bisphenol A, endocrine disruptor, endocrine-disrupting chemical, estrogen receptor binding, estrogenic activity, plastic

## Abstract

Background: Chemicals having estrogenic activity (EA) reportedly cause many adverse health effects, especially at low (picomolar to nanomolar) doses in fetal and juvenile mammals.

Objectives: We sought to determine whether commercially available plastic resins and products, including baby bottles and other products advertised as bisphenol A (BPA) free, release chemicals having EA.

Methods: We used a roboticized MCF-7 cell proliferation assay, which is very sensitive, accurate, and repeatable, to quantify the EA of chemicals leached into saline or ethanol extracts of many types of commercially available plastic materials, some exposed to common-use stresses (microwaving, ultraviolet radiation, and/or autoclaving).

Results: Almost all commercially available plastic products we sampled—independent of the type of resin, product, or retail source—leached chemicals having reliably detectable EA, including those advertised as BPA free. In some cases, BPA-free products released chemicals having more EA than did BPA-containing products.

Conclusions: Many plastic products are mischaracterized as being EA free if extracted with only one solvent and not exposed to common-use stresses. However, we can identify existing compounds, or have developed, monomers, additives, or processing agents that have no detectable EA and have similar costs. Hence, our data suggest that EA-free plastic products exposed to common-use stresses and extracted by saline and ethanol solvents could be cost-effectively made on a commercial scale and thereby eliminate a potential health risk posed by most currently available plastic products that leach chemicals having EA into food products.

Chemicals that mimic or antagonize the actions of naturally occurring estrogens are defined as having estrogenic activity (EA), which is the most common form of endocrine disruptor activity [Interagency Coordinating Committee on the Validation of Alternative Methods (ICCVAM) 2003, 2006; National Research Council 1999]. Chemicals having EA typically interact with one or more of the classical nuclear estrogen receptor (ER) subtypes: ERα, ERβ, or nonclassical membrane or ER-related subtypes (Hewitt et al. 2005; Matsushima et al. 2008; National Research Council 1999). In mammals, chemicals having EA can produce many health-related problems, such as early puberty in females, reduced sperm counts, altered functions of reproductive organs, obesity, altered sex- specific behaviors, and increased rates of some breast, ovarian, testicular, and prostate cancers (Della Seta et al. 2006; Gray 2008; Kabuto et al. 2004; National Research Council 1999; Newbold et al. 2004; Patisaul et al. 2006, 2009). Fetal, newborn, and juvenile mammals are especially sensitive to very low (sometimes picomolar to nanomolar) doses of chemicals having EA (Gray 2008; vom Saal et al. 2005). Many of these effects observed in mammals are also expected to be produced in humans, because basic endocrine mechanisms have been highly conserved across all classes of vertebrates (Kavlock et al. 1996; National Research Council 1999).

Thermoplastics, which are used for many items that contain food, are made by polymerizing a specific monomer or monomers in the presence of catalysts into a high-molecular- weight chain known as a thermoplastic polymer [see Supplemental Material, Figure 1 (doi:10.1289/ehp.1003220)]. The resulting polymer is mixed with small quantities of various additives (antioxidants, plasticizers, clarifiers, etc.) and melted, mixed, extruded, and pelletized to form a base thermoplastic resin. Base resins are either used as is [e.g., bisphenol A (BPA)-based polycarbonate (PC), non-BPA-based polypropylene (PP) copolymer (PPCO), and non-BPA-based PP homopolymer (PPHO)] or, more commonly, mixed with other resins, additives, colorants, and/or  extenders to form plastic compounds (e.g., polymer blends and precolored polymers). Plastic products are then made by using one or more plastic compounds or resins to form a finished plastic part that can be subjected to finishing processes that may use inks, adhesives, and so forth, to make a finished product.

**Figure 1:**
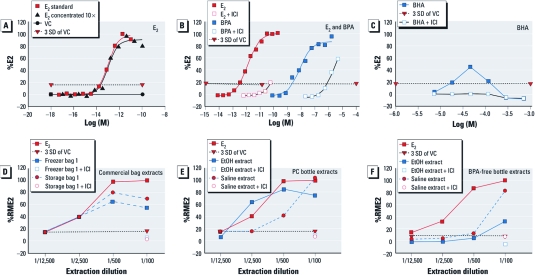
Results of MCF‑7 assays shown as dilution response curves (%E2) for E2 (*A*), E2 and BPA (*B*), BHA (*C*), and %RME2 of extracts of plastic bags (*D*), a PC bottle (*E*), and a BPA-free bottle made from PETG (*F*). Abbreviations: PETG, PET glycol-modified polyethylene terephthalate; VC, vehicle control. Dotted lines represent 3 SD from the response. In *B–F*, the negative control (1% EtOH or saline) equals 0% E2. The E2 standard (10^–9^ M) is the positive control diluted as indicated in *C–F*. Each point plotted is the average of three or four replicates for each concentration whose SD is very small and falls within the space taken up by each data point. In (*A*), E2 was dissolved in EtOH (standard extract) or concentrated 10× and rediluted to show that the EtOH concentration protocol has very little effect on the EC_50_ of E2 (50% E2). The EC_50_ of E2 is approximately 1.3 × 10^–13^ M, and the threshold of detection (15% E2) is approximately 10^–15^ M. The maximum E2 response was attained at 10^–11^ M and remained constant at higher E2 concentrations. (*B*) The EC_50_ of both E2 (as in *A*) and BPA is approximately 6.6 × 10^–8^ M, and threshold detection is approximately 10^–9^ M, all suppressed by 10^–8^ M ICI. (*C*) BHA does not meet criteria needed for accurate calculation of EC_50_ [see Supplemental Material, pp. 5–7 (doi:10.1289/ehp.1003220)]. EA is positive; its maximum response is about 50% E2 (i.e., 50% RME2) and is suppressed by 10^–8^ M ICI. In *D*, commercially available plastic bags were extracted by 100% EtOH. Commercially available PC (*E*) and BPA-free (*F*) bottles were extracted with saline or EtOH as indicated.

As previously described (Begley et al. 1990, 2005; De Meulenaer and Huyghebaert 2004), plastic resins and manufacturing protocols [see Supplemental Material, Figure 1 (doi:10.​1289/ehp.1003220)] collectively use many monomers and additives that may exhibit EA because they have physicochemical properties, often from an insufficiently hindered phenol (HP) group, that enable them to bind to ERs (see Supplemental Material, Table 1). Because polymerization of monomers is rarely complete and additives are not chemically part of the polymeric structure, chemicals having EA can leach from plastic products at very low (e.g., nanomolar to picomolar) concentrations that individually or in combination can produce adverse effects, especially in fetal to juvenile mammals. This leaching of monomers and additives from a plastic item into its contents is often accelerated if the product is exposed to common-use stresses such as ultraviolet (UV) radiation in sunlight, microwave radiation, and/or moist heat via boiling or dishwashing. The exact chemical composition of almost any commercially available plastic part is proprietary and not known. A single part may consist of 5–30 chemicals, and a plastic item containing many parts (e.g., a baby bottle) may consist of ≥ 100 chemicals, almost all of which can leach from the product, especially when stressed. Unless the selection of chemicals is carefully controlled, some of those chemicals will almost certainly have EA, and even when using all materials that initially test EA free, the stresses of manufacturing can change chemical structures or create chemical reactions to convert an EA-free chemical into one with EA.

**Table 1:** Percentage of unstressed plastic products having EA in at least one extract.

Table 1. Percentage of unstressed plastic products having EA in at least one extract.																
		Extraction solvent														
		EtOH				Concentrated EtOH				Saline				Any extract		
Plastic product		*n*		%D		*n*		%D		*n*		%D		*n*		%D
Resin type																
HDPE		13		69		11		55		18		56		30		70
PP		23		52		6		33		16		81		37		68
PET		30		40		17		94		34		76		57		75
PS		13		62		—		—		16		38		28		50
PLA		10		70		1		100		8		100		11		91
PC		1		0		1		100		2		100		2		100
Product type																
Flexible packaging		82		66		6		33		35		74		121		67
Food wrap		9		100		—		—		9		78		9		100
Rigid packaging		57		56		18		67		31		45		83		64
Baby bottle component		13		69		—		—		16		94		19		89
Deli containers		11		36		—		—		7		7		16		44
Plastic bags		33		97		1		100		23		96		43		98
Product retailer																
Large retailer 1		31		81		2		100		4		75		36		81
Large retailer 2		4		50		4		0		50		54		53		53
Large retailer 3		18		83		2		100		7		29		25		72
Large retailer 4		37		51		—		—		—		—		37		51
Large retailer 5		20		50		3		100		4		100		23		70
Organic retailer 1		28		71		5		60		5		80		32		81
Organic retailer 2		33		88		1		100		10		80		35		89
Total for extract		308		68		51		73		214		69		455		72
Abbreviations: —, not tested; %D, percent detectable (extract produced cell proliferation > 15% RME2; see “Materials and Methods”); *n*, total number of samples purchased (less than the sum of *n* values for individual extracts if some items were tested by more than one extraction protocol); PLA, polylactic acid. Data are percentages of samples for which EA was detected using a standard or concentrated EtOH extract, a saline extract, or one or more such extracts (any extract). Some individual items are listed in two or three categories (e.g., PET and baby bottles) but were counted only once for the extract total. Baby bottle components comprised 11 bottles and 2 sealant ring components.																

Very few studies (Soto et al. 1991; Till et al. 1982) have examined the extent to which plastics that presumably do not contain BPA nevertheless release other chemicals having detectable EA. For example, a recent comprehensive review [table on page 72 of Gray (2008)] described polyethylene (PE), PP, and PE terephthalate (PET) plastics as being “‘OK’ for use with respect to release of chemicals exhibiting EA.”

Here, we report that most of the > 500 commercially available plastic products that we sampled—even those that are presumably BPA free—release chemicals having detectable EA, especially if they are assayed by more polar and less polar solvents and exposed to common-use stresses. That is, we show that, to reliably detect such leachable chemicals having EA, unstressed or stressed plastic resins or products should be extracted with more polar (e.g., saline) and less polar [e.g., ethanol (EtOH)] solutions and exposed to common-use stresses (boiling water, microwaving, and UV radiation).

## Materials and Methods

We developed a sensitive and accurate roboticized version of the MCF-7 cell proliferation assay (E-SCREEN assay) that has been used for decades to reliably assess EA and anti-EA (Leusch et al. 2010; Soto et al. 1995) and is currently undergoing validation for international use by ICCVAM/NTP (National Toxicology Program) Interagency Center for the Evaluation of Alternative Toxicological Methods (NICEATM). Chemicals with EA bind to ERs (ERα, ERβ, or ER-related subtypes) and activate the transcription of estrogen-responsive genes, which leads to proliferation of MCF-7 cells.

Detailed methods for the MCF-7 assay are provided in Supplemental Material, (doi:10.1289/ehp.1003220). In brief, plastic resins or products were extracted using saline, a more polar solvent, or EtOH, a less polar solvent. Aliquots of the extracts were then diluted four to eight times to produce up to eight test concentrations. Each test chemical or extract at each concentration was added in triplicate or quadruplicate to 96-well plates containing MCF-7 cells in EA-free culture media. After 6 days of exposure, the amount of DNA per well, an indication of cell proliferation, was assayed using a microplate modification of the Burton diphenylamine assay (Burton 1956; Natarajan 1994).

The effect of a test chemical or extract on proliferation was expressed as the %E2, a percentage of the maximum DNA per well produced by the maximum response to 17β-estradiol (E2; positive control) corrected by the DNA response to the vehicle (negative) control [see Supplemental Material, Equation 1 (doi:10.1289/ehp.1003220)]. For estrogenic test chemicals, the concentration needed to obtain half-maximum stimulation of cell proliferation [half-maximal effective concentration (EC_50_), a measure of binding affinity] was calculated from best fits to dose–response data that meet a well-defined set of criteria by Michaelis-Menton kinetics. The estrogenicity of extracts was calculated as the relative maximum %E2 (%RME2; a measure of response amplitude), a percentage of the maximum DNA per well produced by an extract at any dilution with respect to the maximum DNA per well produced by E2 at any dilution, corrected by the DNA response to the vehicle (negative) control (see Supplemental Material, Equation 2). If a test chemical had a positive response (> 15% RME2) but an EC_50_ could be calculated because not all criteria were met, then the estrogenicity of the test chemical was characterized simply as EA positive or by its %RME2.

The EA of a test chemical or extract was considered detectable if it produced cell proliferation > 15% of the maximum response to E2 (> 15% RME2), which is > 3SDs from the historic control baseline response (about 10^–15^ M), which is a rather conservative measure of EA detectability. Stimulation of MCF-7 proliferation induced by the test chemical or extract was confirmed to be estrogenic (compared with nonspecific) in an EA confirmation study: If the stimulation of MCF-7 proliferation by a test chemical or extract was suppressed by coincubation with a strong antiestrogen [ICI 182,780 (ICI) at 10^–7^ to 10^–8^ M], the EA of the test chemical or extract was confirmed. Therefore, a test chemical or extract was classified as not having detectable EA if it did not induce MCF-7 cell proliferation or if it induced proliferation that could not be inhibited by ICI.

Figure 1 shows typical MCF-7 responses plotted as %E2. Figure 1A–C show responses to some test chemicals: E2 (positive control), BPA, and butylated hydroxyanisole (BHA; a common antioxidant). Figure 1D–F show %RME2 responses to test extracts of plastic food bags, PC bottles, and BPA-free baby bottles and their ICI-suppressed responses, confirming their EA. Some chemicals or products were also analyzed for anti-EA [for details, see Supplemental Material, pp. 7–8 (doi:10.1289/ehp.1003220)].

*Purchase and analyses of plastic products in survey studies.* For Tables 1 and 2, we purchased 455 plastic products used to contain foodstuffs from various commercial retailers from 2005 through 2008. The relative frequency of products having detectable EA did not change with later compared with earlier purchases. In some cases, we instructed undergraduate students or employees to purchase a mix of plastic items used to contain foodstuffs from a given large retailer (Albertsons, H-E-B, Randalls, Target, Wal-Mart, Trader Joe’s, and Whole Foods) mainly in the Austin, Texas, or Boston, Massachusetts, areas, some of which market many “organic” products. In other cases, we purchased products of a particular plastic type (e.g., PE- or PP-based containers). We recorded the retailer, resin type [high-density PE (HDPE), PET, PC, PP, polystyrene (PS), polylactic acid], and product type (flexible packaging, food wrap, rigid packaging, baby bottle component, deli containers, plastic bags). In addition, because the contents of some plastic items might have added or extracted chemicals having EA from the plastic containers before we purchased and tested the products (Sax 2010), we recorded whether the plastic items had contents or were empty when purchased. For any plastic container having contents, we thoroughly washed out the container with distilled water before testing the plastic. Except for PC-based items, none of these products were known to contain BPA. (Plastic products typically do not list their chemical composition, which is proprietary to the manufacturer.) Samples were chosen in product areas where adverse health effects might occur if the samples leached chemicals having EA. Samples from each retailer generally included most of the product types listed above. In addition to surveying commercially available products, we tested plastic resins [e.g., PC, PET, glycol-modified PET (PETG)] that were purchased from M. Holland Company (Northbrook, IL) and individual chemicals used to manufacture plastic products [e.g., BPA, BHA, butylated hydroxytoluene (BHT), dimethyl terephthalate, etc.] that were purchased in their purest form from Sigma-Aldrich (St. Louis, MO).

**Table 2:** Percentage of unstressed plastic products having detectable EA (> 15% RME2) in two extracts.

Table 2. Percentage of unstressed plastic products having detectable EA (> 15% RME2) in two extracts.										
				Extraction solvent						
Category		*n*		EtOH only		Saline only		Both EtOH and saline		Either EtOH or saline
HDPE		13		15		31		15		61
PET		21		19		29		52		100
PP		4		0		25		75		100
PLA		7		0		14		86		100
Bottles		38		13		34		42		89
Baby bottles		11		0		36		64		100
Rigid packaging		10		30		20		40		90
Food wrap		8		25		0		75		100
All products		102		17		21		54		92
PLA, polylactic acid. Values shown are percent (%) of unstressed plastic items (*n*) having detectable EA (> 15%RME2) only in an EtOH extract (and not in a saline extract), only in a standard saline extract (and not in an EtOH extract), in both EtOH and saline extracts, or in either EtOH or saline extracts. The last column is the sum of the three previous columns. “All products” is the total for each column when each product (*n* = 102) is only counted once (some products are listed in two categories). The standard EtOH extract was used for most (*n* = 81) products and the concentrated EtOH extract for the remainder (*n* = 21). If EA was detected in a saline or standard EtOH extract in survey studies such as those reported in Table 1, other extracts often were not performed. A concentrated EtOH extract was usually used to generate data shown in Tables 1 and 2 only if EA was not detected in a saline or standard EtOH extract. That is, samples listed for concentrated EtOH in Table 1 and EtOH in Table 2 had a selection bias for not having detectable EA.										

Many plastic products have more than one plastic part. For example, baby bottles have 3–10 different plastic parts in various combinations [bottle, nipple, anticolic item(s), sealing ring(s), liner bag, cap, etc.], each part typically having different and rather unique combinations of 5–30 chemicals. Over the course of this entire study, we assayed > 100 component parts from > 20 different baby bottles, including many advertised as BPA free. Only some (13) of these component parts were purchased for the initial survey study (Tables 1 and 2).

Most of the samples (338 of 455) in the survey study (Tables 1 and 2) were extracted using only one extraction protocol. For the remaining samples (*n* = 102), both saline and EtOH extractions were used so that the efficacy of each protocol could be directly compared. We used a paired Student’s *t*-test to test whether differences between pairs of samples were statistically significant (*p* < 0.05).

**Figure 2:**
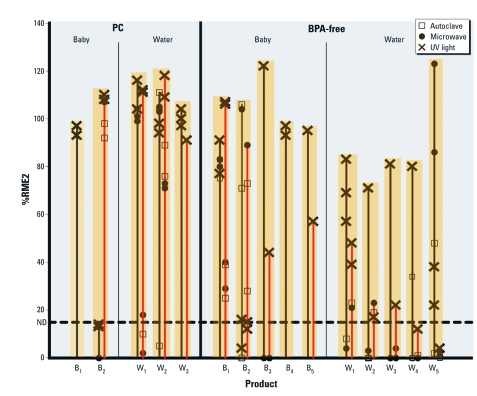
Total EA released by some PC and BPA-free water bottles (W) and baby bottles (B). The leaching of chemicals having EA (measured as %RME2; excluding caps, nipples, and other components) were extracted using saline or EtOH as solvents and exposed to autoclaving, microwaving, and/or UV light (see “Materials and Methods” for details). BPA-free water bottles W_1_, W_2_, W_3_, and W_4_ are PETG, and W_5_ is PET. BPA-free baby bottles B_1_ and B_2_ are polyethersulfone; B_3_ is PETG; and B_4_ and B_5_ are PP. Orange bars indicate the data set for each individual product. The %RME2 for saline extracts is represented by solid black lines and for EtOH as solid red lines. Symbols represent the %RME2 of chemicals released by each assay of a product after an autoclaving stress, microwaving stress, and UV light stress (see figure key). The dotted horizontal line at 15% RME2 is the rather conservative value below which EA was considered nondetectable (ND) for any assay. For some products shown (e.g., PC B_1_, BPA-free B_4_), if one solvent and/or stress condition showed reliably detectable EA, other solvents and stress conditions were not subsequently tested. Some values plotted as 0% RME2 actually had slightly negative %RME2 values (–1% to –7% RME2) due to cellular toxicity.

*Protocols for common-use stresses of some plastic items.* Given that common-use stresses can alter the complex chemical composition of plastics and/or increase the rate of leaching (Begley et al. 1990, 2005; De Meulenaer and Huyghebaert 2004), for some resins or products, we examined how leaching of chemicals having EA might be affected by exposure to microwave radiation, autoclaving (moist heat), and UV light. Additional plastic items, some of which are described in Figure 2 and Table 3, were purchased in 2008–2010 and subjected to common-use stresses. In addition, we tested a variety of resins (including PE- and PP-based resins; Table 3), antioxidants [see Supplemental Material, Table 3 (doi:10.1289/ehp.1003220)], and other additives or processing agents (see Supplemental Material, Table 4) identified by our laboratory as being free of detectable EA and hence possibly suitable for use to produce final products that would be EA free even after exposure to common-use stresses.

**Table 3:** Representative %RME2 values for stressed resins or parts made from flexible or HC polymers.

Table 3. Representative %RME2 values for stressed resins or parts made from flexible or HC polymers.												
		Stress/extraction solvent										
		Microwave				UV				Autoclave		
Sample type		Saline		EtOH		Saline		EtOH		Saline		EtOH
Flexible polymers												
LDPE resin 1		5		7		0		4		4		30*a*
LDPE resin 2		3		7		26*a*		3		–1		27*a*
PET water bottle		100*a*		3		31*a*		2		47*a*		1
LDPE resin P1		2		3		0		0		4		5
HDPE resin P2		6		–4		2		–2		–1		–3
PPHO resin P3		0		–4		3		2		–6		–3
PPCO resin P4		3		7		–7		–6		–9		–3
HDPE resin P5		ND		ND		ND		47*a*		ND		ND
HC polymers												
Water bottle 1.1		3		23*a*		71*a*		17*a*		–1		19*a*
Water bottle 1.2		4		21*a*		57,*a* 69,*a* 98*a*		48,*a* 39*a*		8		23*a*
Water bottle 2.1		–7		–5		81*a*		22*a*		0		4
Water bottle 2.2		34*a*		–2		80*a*		12		–1		1
PETG baby bottle		0		–2		122*a*		44*a*		0		1
PETG resin 1		–8		17*a*		61*a*		111*a*		0		15*a*
PS 1		4		3		17*a*		45*a*		76*a*		0
COC 3		9		7		20*a*		20*a*		0		6
COC resin P18		4		1		9		11		1		–2
COC resin P19		6		2		6		–2		4		2
Abbreviations: COC, cyclic olefin copolymer; ND, not determined; PPCO, polypropylene copolymer; PPHO, polypropylene homopolymer. Numerical values are %RME2 responses of extract for several different baby bottle and other component parts. Resins designated with P (e.g., P1, P18) are EA-free formulations developed at PlastiPure. Resin P5 exhibited EA when stressed. Multiple values for water bottle 1.2 under UV stress are data for repeated analyses. **a**Plastic items leaching chemicals having detectable EA > 15% RME2.												

We used the following stresses:

- Samples were placed about 2 feet from a 254-nm fluorescent fixture for 24 hr, simulating repeated UV stress by sunlight (e.g., water bottles) or UV sterilizers (e.g., baby bottles and medical items)
- Samples were autoclaved at 134°C for 8 min, simulating moist heat stress in an automatic dishwasher
- We heated samples in a microwave 10 times for 2 min each, using a 1,000-W kitchen microwave oven set to “high,” simulating heat and microwave radiation stress to reusable food containers.

## Results

*Release of chemicals having EA from unstressed plastics.* Tables 1 and 2 show the percentage of samples in each category that had reliably detectable EA (> 15% RME2) in our survey of 455 commercially available plastic products. [For the %RME2 and content status of individual samples, as well as the average %RME2 for products classified by resins (HDPE, PP, PET, PS, polylactic acid, PC), product type (flexible packaging, food wrap, rigid packaging, baby bottle components, plastic bags), and retailer (large retailers 1–5 and large organic retailers 1 and 2), see Supplemental Material, Table 5 (doi:10.1289/ehp.1003220).] For example, 9 of 13 HDPE plastic products extracted by our standard EtOH protocol (69%) had detectable EA (Table 1), with a %RME2 (mean ± SD) of 66% ± 25% (see Supplemental Material, Table 5A). For PET products extracted by saline, 26 of 34 (76%) had detectable EA (Table 1) with a %RME2 of 64% ± 41% (see Supplemental Material, Table 5C). We found no consistent correlation between the percentage of items in a product type with detectable EA and their mean %RME2 (data not shown).

We found no significant difference (*p* > 0.05) in the percentage of items with detectable EA between those with contents and those with no contents (76%, *n* = 160) at the time of purchase based on the standard EtOH extraction protocol [67% vs. 70%; see Supplemental Material, Table 2A (doi:10.1289/ehp.1003220)], the standard saline protocol (62% vs. 75%; see Supplemental Material, Table 2C), or all extraction protocols combined (69% vs. 76%). Most important, items with no contents in all categories exhibited detectable EA in at least one protocol (see Supplemental Material, Tables 2 and 5), including 78% of items made from HDPE (*n* = 18), 57% from PP (*n* = 14), and 100% from PET (*n* = 6). Given all of these results, we present the data for all items shown in Tables 1 and 2 without regard to their content status.

*Using different solvents increased the probability of detecting EA.* Most (71%) unstressed plastic items released chemicals with reliably detectable EA in one or more extraction protocols, independent of resin type, product type, or retailer (Table 1). Results often differed between saline and EtOH extracts of the same unstressed plastic item, and EA was reliably detected most frequently (92% of all items listed in Table 2) when analyzed using both saline (more polar) and EtOH (less polar) extracts. For example, 15% of unstressed HDPE plastic items leached chemicals with detectable EA into both EtOH and saline extracts, 15% leached only into EtOH, and 31% leached only into saline (Table 2). That is, the leaching of a chemical with EA was significantly (*p* < 0.01) more likely to be detected if we used both polar and nonpolar solvents (61%) than if we used only one solvent (30% for EtOH only or 45% for saline only). We obtained similar results for all types of plastic products (data not shown).

Assays of > 100 component parts from > 20 different baby bottles, including many advertised as BPA free, indicated that extracts of at least one bottle component of each baby bottle always had EA based on at least one assay (some data shown in Table 2 and Figure 2), as did at least one other component part (data not shown).

*Stresses increased the release of chemicals having EA.* Leaching of chemicals with EA was increased by common stresses. For example, one unstressed sample of an HDPE resin (P5 in Table 3) that had no detectable EA (i.e., RME2 < 15%) in two saline extracts and two EtOH extracts released chemicals with EA equivalent to 47% RME2 when extracted using EtOH after the resin was stressed with UV light. Similarly, two samples of low-density PE resins (LDPE resins 1 and 2) and PETG resins (PETG baby bottle and PETG resin 1) that had no detectable EA before stressing subsequently exhibited EA when stressed, especially by UV (Table 3). Samples (*n* > 10) of products made from PETG resins advertised as BPA free all released detectable EA when stressed, especially by UV light. Similarly, 25% of unstressed samples of PET and 50% of unstressed PS products surveyed did not have detectable EA in assays of EtOH and/or saline extracts (Table 1). However, when stressed and assayed using both saline and EtOH extracts, all PET (*n* > 10) and PS (*n* > 10) products released chemicals having detectable EA in at least one extracting solvent (Table 3).

**Figure 3:**
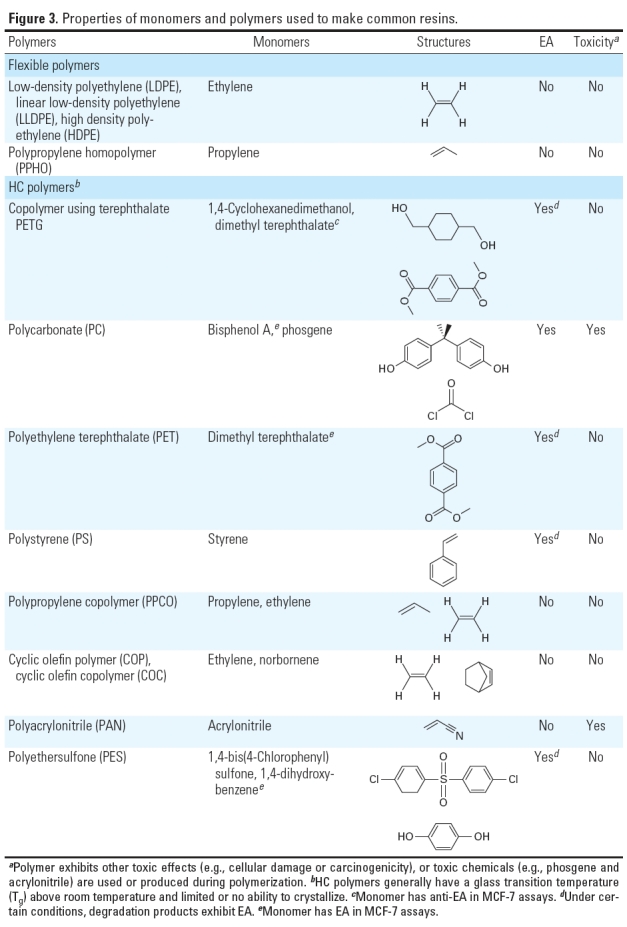
Properties of monomers and polymers used to make common resins.

*EA-containing and EA-free monomers.* Polymerization of monomers is rarely complete, and unpolymerized monomers are almost always released from polymer resins (Begley et al. 1990, 2005; De Meulenaer and Huyghebaert 2004). PE and PP polymers are often used to manufacture flexible and/or  nontransparent rigid products (Figure 3). MCF-7 assays (*n* = 6) consistently showed that extracts of “barefoot” (no additives) polymers (e.g., LDPE resin P1 in Table 3) were EA free, even when stressed. (PP-based polymers require antioxidants to prevent severe degradation during their use in manufacturing plastic products.) Furthermore, PE- and PP-based resins containing appropriate additives to produce fit-for-use products could be constructed that remained EA free (*n* > 100 assays of > 10 resins), even when exposed to common-use stresses. Representative data from several such resins (LDPE resin P1, HDPE resin P2, PP homopolymer resin P3, PP copolymer resin P4) are shown in Table 3.

Figure 3 also shows other monomers and polymers that can or cannot be used to make hard-and-clear (HC) plastics. For example, HC PC plastics (*n* > 10) all released chemicals having EA (e.g., PC baby bottle B_1_ and PC water bottle W_1_ in Figure 2), almost certainly phenolics such as BPA (Figure 1B). The dimethyl terephthalate monomer used to make PET and PETG plastics exhibited anti-EA (*n* = 3 assays; data not shown; for anti-EA assay protocol, see Supplemental Material (doi:10.1289/ehp.1003220)]. Furthermore, breakdown products of dimethyl terephthalate, PET, and PETG resins probably contain and release phenolic moieties that have EA that account for some of the data for PET products in Tables 1 and 2. Polyethersulfone HC products also consistently released chemicals having EA or anti-EA, especially when stressed with UV light (data not shown), possibly from unreacted phenolic monomer residues or phenolic stress-degradation products. In contrast, some HC cyclic olefin polymer/cyclic olefin copolymer polymers produced from saturated cyclic olefin monomers contained no phenolics and did not release chemicals having detectable EA, even when stressed (Table 3).

Polymers that can be made EA free have a similar cost compared with polymers made from monomers that have EA. For example, currently, clarified PP having no additives that exhibit EA (even when stressed) that is suitable for molding bottles costs approximately $1.20/lb. PP resins containing additives that have EA also cost about $1.20/lb. Commodity resins such as PET, which are made from monomers having EA and are suitable for molding bottles, are priced at approximately $1.28/lb (Plastics News 2011).

*EA-containing and EA-free additives.* Many additives are physically, but not chemically, bound to a polymeric structure and hence can almost always leach from the polymer, especially when stressed (Begley et al. 1990, 2005; De Meulenaer and Huyghebaert 2004). Antioxidants are the most critical class of additives because they prevent or minimize plastic degradation due to oxidation that breaks polymer chains (chain scission) and/or causes cross-links (Kattas et al. 2000). The oldest and most common antioxidants deemed suitable for food contact belong to a chemical class known as HPs (hindered phenols), such as BHT and BHA, in large part because both are inexpensive and assumed to be nontoxic. However, BHT (*n* = 4 assays) had reliably detectable EA, as did BHA (*n* = 3 assays). [The EC_50_ of BHT and BHA (Figure 1C) could not be accurately calculated because both also exhibited cellular toxicity at higher concentrations (10^–5^ M).] Other commonly used HP antioxidants (*n* = 4/5) and organophosphines (*n* = 6/7) also exhibited reliably detectable EA, especially when exposed to moist heat, which presumably causes hydrolysis (data not shown). For example, proprietary antioxidants Phos (phosphate) OX 1 and HP AOX 2 had no detectable EA, whereas HP AOX 1 and Ph (bisphenol) AOX 1 had reliably detectable EA [see Supplemental Material, Table 3 (doi:10.1289/ehp.1003220)].

Many other additives (*n* > 50) with a phenolic group had reliably detectable EA, such as agents found in many base resins [tris(nonylphenyl) phosphite, octylphenol, nonylphenol, butylbenzene phthalate], colorants (especially blues or greens with phthalocyanine groups), PS-based purge compounds, and mold-release agents [see Supplemental Material, Table 4 (doi:10.1289/ehp.1003220)]. In contrast, many metal- oxide–based inorganic pigments did not exhibit EA. However, these EA-free pigments are often mixed with dispersing agents and carrier resins that have EA to produce colorant masterbatch concentrates. Nevertheless, we have identified resins, dispersants, pigments, and antioxidants that are approved by the Food and Drug Administration for direct food contact (see Supplemental Material, Tables 3 and 4) to create colorant masterbatch concentrates (*n* > 100) that produce even colorant dispersion into plastics and that have no detectable EA, cellular toxicity, or adverse processing effects, even when stressed.

Because additives comprise a small fraction (typically 0.1–1% by weight) of plastic resins and compounds and because plastic resins and compounds using EA-free additives are processed during manufacture in a nearly identical manner as conventional resins and compounds containing chemicals with EA, the replacement of additives having EA with EA-free additives should have very little impact on the cost of the final product. Furthermore, EA-free additives have only a slightly higher or no additional cost compared with additives with EA, so that their cost impact is very small or nonexistent.

*Products currently marketed as BPA free are not EA free.* In response to market and regulatory pressures to eliminate BPA in HC plastics, BPA-free HC materials have recently been introduced as replacements for PC resins. PET and PETG are two such resins, but HC plastic products made from these resins leached chemicals that had detectable EA (Tables 1–3, Figures 2 and 3), often in the absence of exposure to common-use stresses. Two popular brands of water bottles made from a PETG resin now marketed as an HC BPA-free replacement also released chemicals having significant EA (W1, W2, W3, and W4; Table 3, Figures 2 and 3), as did uncompounded PETG resins (Table 3). Most PE/PP-based plastic products were presumably BPA free but nevertheless had readily detectable EA (Tables 1 and 2), almost certainly due to one or more additives having EA. Many components of BPA-free baby bottles had reliably detectable EA (22–95% RME2) when extracted in either saline or EtOH, including the bottle, nipple, anticolic device, and liner (data not shown).

In fact, all BPA-replacement resins or products tested to date (*n* > 25) released chemicals having reliably detectable EA (data not shown), including polyethersulfone and PETG, sometimes having more total EA measured as %RME2 than many PC products when stressed. For example, the %RME2 released by various BPA-free baby and water bottle component parts extracted by saline or EtOH solutions and exposed to one or more common-use stresses can be greater than PC products under the same conditions (Figure 2). UV stress, in particular, often leads to the release of chemicals having greater EA than BPA-containing HC plastics currently sold. For example, saline extracts of BPA-free baby bottle B_3_ (Figure 2) after exposure to UV showed greater EA than did any of the PC baby bottle extracts after any of the stresses. Saline extracts from BPA-free baby bottle B_1_ after any of the stresses (microwave, autoclave, or UV) showed greater EA than did the saline extracts from PC baby bottle B_2_ after any of the stresses. EtOH extracts from BPA-free baby bottle B_1_ after UV stress showed greater EA than extracts from PC baby bottle B_1_. Saline extracts from BPA-free baby bottle B_2_ after microwave or autoclave stresses showed greater EA than did saline extracts from PC baby bottles B_1_ or B_2_ after any of the stresses. Note also in Figure 2 that multiple extracts of the same product using the same solvent/stress combination typically gave rather similar %RME2 data, but different solvent/stress combinations gave very different results, from very high EA to nondetectable EA. For example, EtOH extracts from PC baby bottle B_2_ showed very high EA under all stress conditions, whereas saline extracts of the same bottle under the same stress conditions showed no detectable EA. Hence, to reliably detect EA, plastic resins or products must be extracted with both polar and nonpolar solvents and exposed to common-use stresses.

## Discussion

*Most plastic products release chemicals having EA.* Our data show that both more polar (e.g., saline) and less polar (e.g., EtOH) solvents should be used to extract chemicals from plastics because the use of only one solvent significantly reduces the probability of detecting chemicals having EA. The ability to detect more polar and less polar chemicals having EA is important because plastic containers may hold either type of liquid or a liquid that is a mixture of more polar and less polar solvents (e.g., milk). When both more polar and less polar solvents are used, most newly purchased and unstressed plastic products release chemicals having reliably detectable EA independent of the type of resin used in their manufacture, type of product, processing method, retail source, and whether the product had contents before testing. However, the lack of significant difference in average percentage having detectable EA between plastic items with and without contents does not imply that the contents do not affect the total EA or specific chemicals having EA released by individual plastic items.

Our data show that most monomers and additives that are used to make many commercially available plastic items exhibit EA. Even when a “barefoot” polymer (no additives) such as PE or polyvinyl chloride does not exhibit EA, commercial resins and products from these polymers often release chemicals (almost certainly additives) having EA.

We found that exposure to one or more common-use stresses often increases the leaching of chemicals having EA. In fact, our data suggest that almost all commercially available plastic items would leach detectable amounts of chemicals having EA once such items are exposed to boiling water, sunlight (UV), and/or microwaving. Our findings are consistent with recently published reports that PET products release chemicals having EA (Wagner and Oehlmann 2009) and that different PET products leach different amounts of EA. For example, different PET products release different amounts of EA measured as %E2 or %RME2 [see Supplemental Material, Table 5C (doi:10.1289/ehp.1003220)], almost certainly because different PET copolymer manufacturers choose different monomers, additive packages, and synthetic processes to produce PET copolymer resins.

Our data are consistent with the hypotheses that the presence of a phenolic moiety is the best predictor of whether a chemical exhibits EA and that benzene moieties often probably convert to phenolic moieties when the monomer and/or polymer is exposed to one or more manufacturing or common-use stresses. For example, although in theory most organophosphites (antioxidants commonly used with HPs to provide synergistic oxidation protection) in their unaltered state should not bind to ERs [see Supplemental Material, Table 1 (doi:10.1289/ehp.1003220)], organophosphites are hydrolytically unstable and often produce phenols when exposed to water (Kattas et al. 2000). Most organophosphite antioxidants we tested exhibited detectable EA (data not shown).

Likewise, various additives that are high-molecular-weight HPs do not have EA, but if exposed to moist heat they can undergo hydrolysis and produce lower-molecular-weight phenolics that have EA. Therefore, antioxidants and other additives should be tested for EA both in their original, unstressed form and after stressing. We can identify monomers and additives (antioxidants, clarifiers, slip agents, colorants, inks, etc.) having no detectable EA for use at all stages of manufacturing processes to make flexible nontransparent or HC plastic items that are EA free, even after exposure to common-use stresses. All of our data suggest that, when both are manufactured in comparable quantities, carefully formulated EA-free plastic products could have all the fit-for-use properties of current EA-releasing products at minimal additional cost.

*BPA free is not EA free.* Although most items listed in Tables 1–3 would not be expected to contain BPA, nevertheless almost all stressed plastic items tested leached chemicals having reliably detectable EA measured as %RME2 if extracted with both more polar and less polar solvents. In response to market and regulatory pressures, BPA-free PET or PETG resins and products have recently been introduced as replacements for PC resins. However, all such replacement resins and products tested to date release chemicals having EA (measured as %RME2), sometimes having more EA than BPA-containing PC resins or products, especially when stressed by UV light (Figure 2, Table 3). Monomer or polymer breakdown products that have EA account for some of this EA, but the rest of the measured EA is almost certainly due to release of additives having EA in BPA-free products, including the bottle and many component parts of baby bottles advertised as BPA free.

*Avoiding a potential health problem.* We recognize that we quantitatively measured EA relative to E2 (EC_50_ or %RME2) using sensitive assay and extraction protocols. Furthermore, it is almost impossible to gauge how much EA anyone is exposed to, given such unknowns as the number of chemicals having EA, their relative EA, their release rate under different conditions, and their metabolic degradation products or half-lives *in vivo*. In addition, the appropriate levels of EA in males versus females at different life stages are currently unknown. Nevertheless, *a*) *in vitro* data overwhelmingly show that exposures to chemicals having EA (often in very low doses) change the structure and function of many human cell types (Gray 2008); *b*) many *in vitro* and *in vivo* studies document in detail cellular/molecular/systemic mechanisms by which chemicals having EA produce changes in various cells, organs, and behaviors (Gray 2008); and *c*) recent epidemiological studies (Gray 2008; Koch and Calafat 2009; Meeker et al. 2009; Swan et al. 2005; Talsness et al. 2009; Thompson et al. 2009) strongly suggest that chemicals having EA produce measurable changes in the health of various human populations (e.g., on the offspring of mothers given diethylstilbestrol, or sperm counts in Danish males and other groups correlated with BPA levels in body tissues).

Many scientists believe that it is not appropriate to bet our health and that of future generations on an assumption that known cellular effects of chemicals having EA released from most plastics will have no severe adverse health effects (Gray 2008; Talsness et al. 2009; Thompson et al. 2009). Because we can identify existing, relatively inexpensive monomers and additives that do not exhibit EA, even when stressed, we believe that plastics having comparable physical properties but that do not release chemicals having detectable EA could be produced at minimal additional cost.

## Supplemental Material

(428 KB) PDF
