# Supplementary material for: Most Plastic Products Release Estrogenic Chemicals: A Potential Health Problem That Can Be Solved
Source: Environ Health Perspect. 2011 Mar 2;119(7):989–96. doi: 10.1289/ehp.1003220 (PMC3222987; doi:10.1289/ehp.1003220)
Supplement: (428 KB) PDF [file ehp.1003220.s001.pdf]

Supplemental Material

Most Plastic Products Release Estrogenic Chemicals: A Health Problem That Can Be Solved

CZ Yang, SI Yaniger, VC Jordan, DJ Klein, and GD Bittner

[In Supplemental Material, EA means EA and anti-EA]**

Table of Contents

Technical Details of the Robotic MCF-7 Assay 3
Materials and Processes in Plastics Production 8
Monomers and Additives in Plastics........ 13
References........ 19

Supplemental Material: Figure
Supplemental Material, Figure 1: Production of a typical plastic part 9

Supplemental Material: Tables
Supplemental Material, Table 1: Physical-chemical properties of chemicals that affect binding to ERs........ 11
Summary Desriptions of Tables 2A-C........ 14
Supplemental Material, Table 2A: Standard EtOH Extraction Protocol 14
Supplemental Material, Table 2B: Concentrated EtOH Extraction Protocol 15
Supplemental Material, Table 2C: Standard Saline Extraction Protocol........ 16
Supplemental Material, Table 3: EA of some common antioxidants 17
Supplemental Material, Table 4: EA of some chemicals used during conversion and/or finishing processes 18
Summary Descriptions of Tables 5A-T 20
Supplemental Material, Table 5A: %RME2 values for individual samples manufactured using HDPE, and mean %RME2 ± SD for all samples for each extraction protocol 22
Supplemental Material, Table 5B: %RME2 values for individual samples manufactured using PP and mean %RME2 ± SD for all samples for each extraction protocol 23
Supplemental Material, Table 5C: %RME2 values for individual samples manufactured using PET and mean %RME2 ± SD for all samples for each extraction protocol 24
Supplemental Material, Table 5D: %RME2 values for individual samples manufactured using Polystyrene, and mean %RME2 ± SD for all samples for each extraction protocol 26
Supplemental Material, Table 5E: %RME2 values for individual samples manufactured using PLA, and mean %RME2 ± SD for all samples for each extraction protocol 26

Supplemental Material, Table 5F: %RME2 values for individual samples manufactured using PC, and mean %RME2 ± SD for all samples for each extraction protocol 28
Supplemental Material, Table 5G: %RME2 values for individual samples of flexible packaging, and mean %RME2 ± SD for all samples for each extraction protocol 29
Supplemental Material, Table 5H: %RME2 values for individual samples of food wrap, and mean %RME2 ± SD for all samples for each extraction protocol 32
Supplemental Material, Table 5I: %RME2 values for individual samples of rigid packaging, and mean %RME2 ± SD for all samples for each extraction protocol 33
Supplemental Material, Table 5J: %RME2 values for individual samples of baby bottle parts, and mean %RME2 ± SD for all samples for each extraction protocol 35
Supplemental Material, Table 5K: %RME2 values for individual samples of delicatessen containers, and mean %RME2 ± SD for all samples for each extraction protocol 36
Supplemental Material, Table 5L: %RME2 values for individual samples of plastic bags, and mean %RME2 ± SD for all samples for each extraction protocol 37
Supplemental Material, Table 5M: %RME2 values for individual samples of plastic items purchased from a large retailer 1, and mean %RME2 ± SD for all samples for each extraction protocol 39
Supplemental Material, Table 5N: %RME2 values for individual samples of plastic items purchased from large retailer 2, and mean %RME2 ± SD for all samples for each extraction protocol 41
Supplemental Material, Table 5O: %RME2 values for individual samples of plastic items purchased from large retailer 3, and mean %RME2 ± SD for all samples for each extraction protocol 43
Supplemental Material, Table 5P: %RME2 values for individual samples of plastic items purchased from a large retailer 4, and mean %RME2 ± SD for all samples for each extraction protocol 44
Supplemental Material, Table 5Q: %RME2 values for individual samples of plastic items purchased from large retailer 5, and mean %RME2 ± SD for all samples for each extraction protocol 46
Supplemental Material, Table 5R: %RME2 values for individual samples of plastic items purchased from large organic retailer 1, and mean %RME2 ± SD for all samples for each extraction protocol 47
Supplemental Material, Table 5S: %RME2 values for individual samples of plastic items purchased from large organic retailer 2, and mean %RME2 ± SD for all samples for each extraction protocol 49
Supplemental Material, Table 5T: %RME2 values for individual samples of all plastic items and mean %RME2 ± SD for all samples for each extraction protocol 51

Technical Details of the Robotic MCF-7 Assay

Rationale: Chemicals with EA bind to ERs (either α, β, or ERRs) and activate the transcription of estrogen-responsive genes, which leads to proliferation of MCF-7 cells. Anti-EA is measured as an ability to reduce MCF-7 cell proliferation by the natural estrogen, 17β-estradiol (E2). MCF-7 cells are perhaps the most widely used *in vitro* model for studying estrogen action and were derived from human breast cancer cells. In the years since these landmark experiments, MCF-7 cell proliferation has been used as the *in vitro* benchmark ("gold standard") for determining EA or anti-EA and for characterizing the EA** of many environmental chemicals (Leusch et al. 2010; Soto et al. 1995).

Equipment: EpMotion robotic workstations placed in a Labconco Class II Biosafety Hood with a 254nm fluorescent fixture are used to seed cells in 96-well plates, prepare serial dilutions of the test chemicals in tissue-culture medium, change media and prepare 96-well plates for DNA quantification assays. DNA quantification, based on the diphenylamine reaction, is conducted on a Bio-Tek PowerWave X 96-well plate reader spectrophotometer.

Materials: Bovine insulin, calf thymus DNA type I, Hoechst dye 33258, EDTA, Hanks' balanced salt solution (HBSS), and E2 are obtained from Sigma Chemical Company (St. Louis, MO). We use fetal bovine serum to maintain the cells, RPMI Medium 1640 with phenol red for cell maintenance and RPMI medium 1640 without phenol red for EA and anti-EA assays. L-Glutamine, Minimum Essential Medium (MEM) non-essential amino acids, antibiotics, anti-mycotics, and lyophilized trypsin are obtained from Invitrogen (Carlsbad, CA).

Cell preparation: Estrogen sensitive MCF-7 cells were obtained from Dr. V. Craig Jordan, Northwestern University Medical School (now at Georgetown Medical School). Stocks of these estrogen-responsive MCF-7 cells are stored in liquid nitrogen. MCF-7 cells are also maintained at 37°C in RPMI with nonessential amino acids, 10 µg/mL phenol red, 10 mM HEPES, 6 ng/mL insulin, 100 units/mL penicillin, 100 µg/mL streptomycin, and 10% fetal bovine serum (maintenance medium). Because the responsiveness of MCF-7 cells can drift, cells are propagated for 2-3 months and then replaced with cells derived from the primary source kept in liquid N_2 storage. An aliquot of cells maintained at 37°C are grown for two days in phenol-free RPMI media containing nonessential amino acids, 10 mM HEPES, 6 ng/mL insulin, 100 units/mL penicillin, 100 µg/mL streptomycin, 1% charcoal-stripped fetal bovine serum, and 4% charcoal-stripped bovine calf serum (EA-free medium). Using the epMotion 5070 unit, MCF-7 cells are seeded at 5000 cells per well in 0.2 mL of EA-free medium in 96-well cell culture plates. The cells are adapted for 3 days in EA-free medium prior to adding test chemicals.

We routinely test each new batch of charcoal-stripped serum for estrogen-stimulated MCF-7 cell growth. Unless E2-stimulated cell growth is ≥ 4 times the negative control growth in seven days and the cell growth rate is similar for the vehicle control (0.5% EtOH) and ICI 182,780, we do not use that batch of serum.

DNA quantification: Briefly, 60 µL of a 1:5 ratio mixture of 0.16% acetaldehyde and 20% v/v perchloric acid, and then 100 µL of 4% diphenylamine in glacial acetic acid, are added to each well. A standard curve is prepared by adding samples of 0.125 to 3 µg DNA/well in 10 µL of HBSS. After incubating the plate overnight at 37°C, the absorbance is measured as the difference between OD_{595} and OD_{700} in a Bio-Tek EL_x 808 plate reader. The output is converted to µg DNA/well by using a third degree polynomial curve fit of the standard curve; the correlation coefficient of the standard curve fit is typically greater than 0.99. Each robotic assay includes a DNA standard for calibration. DNA absorbance at 1 mg/mL is set at 50 absorbance units. The DNA concentration of MCF-7 cells accurately estimates the number of cells (Taylor et al. 1995). An estimate of cell number/well is obtained by dividing the total amount of DNA per well by 20 pg of DNA per MCF-7 cell.

Treatment of test chemicals or extracts (also see description in text of main paper): A test chemical is dissolved in ethanol (EtOH) and/or culture media (saline) at a stock concentration of 10^{-1}M or to the highest soluble concentration and then diluted 6-12 times in 2-10x steps in EA-free saline.

We cut plastic items to be extracted into 4 mm square pieces so as to standardize extraction methods and make comparisons among plastic items more valid. Cutting the plastic item has only a small effect on the surface area. For example, a typical plastic resin sheet or item has a thickness of 0.05 cm and a polymer density of 1 g/cc, so that the area per gram is 42 cm^2 for 2.5 cm squares. For 4 mm squares, the surface area per gram is 50 cm^2. Additionally, the migration of typical additives (e.g. antioxidants) is a very weak function of surface area - the rate limiting step for most common migrants is the removal of the extracted chemical from the boundary region between the plastic and the extracting solvent (or the rate of hydrolysis of the extracted material in the case of hindered phenolic antioxidants).

Plastic resins or products are extracted by first sterilizing finely cut plastic in a glass test tube placed about two feet from a 254 nm fluorescent fixture in a Labconco Hood for 30 minutes to kill bacteria before adding sterile solvent, sealing the test tube, removing them from the hood and placing them in a 37°C incubator for 72 hours.

The extracts are performed using one of three protocols:

- Culture media (about 0.3M saline) is used as a more polar solvent in a ratio of 1 g plastic/1.5 mL media that, following the extraction period, is then removed from the vial and diluted 2x with EA-free medium containing 2% charcoal-stripped FBS and 8% of charcoal-stripped calf serum as the first test concentration.
- EtOH is used as a less polar solvent in a ratio of 1 g plastic/1 mL and, following the extraction period is then removed from the vial and diluted 100x with culture medium as the first test concentration.
- Some 1 g/ml EtOH extracts are first concentrated 20x and then diluted 100x with culture medium as the first test concentration.

Aliquots of the extracts are then diluted 4-8 times with culture medium typically using a 5x dilution factor to obtain the 2nd through 4th (or up to 8th) test concentrations of the extract.

We add each test chemical or extract at each concentration in triplicate or quadruplicate to 96-well plates containing MCF-7 cells in EA-free culture media for our EA assay or in 10^{-12}M E2 for our anti-EA assay. The position of test and control chemicals on a 96-well plate depends, in part, upon whether or not the test chemical or extract is volatile. Volatile chemicals are tested on a separate 96-well plate to avoid cross-contamination of adjacent wells. We often pre-screen test chemicals and extracts for volatility before performing an EA or anti-EA assay.

Cells in 96-well plates are incubated with test chemicals or extracts and controls at 37°C. The media and its contents are changed every other day for 6 days. At the end of the 6 day exposure, the media is removed, the wells washed once with HBSS, and then assayed to quantify amounts of DNA/well using a microplate modification of the Burton diphenylamine assay (Burton 1956; Natarajan 1994).

EA Confirmation: Estrogenic stimulation (as opposed to nonspecific stimulation) of MCF-7 proliferation (measured as increased amount of DNA per well) induced by the test chemical or extract is confirmed as estrogenic in an EA confirmation study: If the stimulation of MCF-7 proliferation by a test chemical or extract is suppressed by co-incubation with an anti-estrogen (ICI 182,780 at 10^{-7} - 10^{-8}M), the estrogenic activity of the test chemical or extract is then confirmed. Otherwise, the EA of the chemical or extract is NOT confirmed and the stimulation of MCF-7 cell proliferation is not via an ER-mediated mechanism.

Quantitative Calculation of EA: For test chemicals or extracts incubated for six days, the DNA/well produced by any dilution of a test chemical or extract is expressed as a percentage of the maximum DNA/well produced by the maximum response to 17β-estradiol (E2, positive control) corrected by the DNA response to the vehicle (negative) control as given by **Equation 1:**

Equation 1

$$\%E2 = \frac{DNA_{testchemical}(\mu g/well) - DNA_{negativecontrol}(\mu g/well)}{DNA_{E2}(\mu g/well) - DNA_{negativecontrol}(\mu g/well)} \text{ x } 100\%$$

For estrogenic test chemicals, the concentration needed to obtain half-maximum stimulation of cell proliferation (EC50, in M) is calculated from best fits to dose-response data that meet a well-defined set of criteria (see below) by Michaelis-Menton kinetics using GraphPad Prism.

The estrogenicity of extracts is calculated as the relative maximum %E2 (%RME2) defined as a percentage of the maximum DNA/well produced by an extract at any dilution with respect to the maximum DNA/well produced by E2 at any dilution corrected by the DNA response to the vehicle (negative) control as given by **Equation 2**:

Equation 2

$$\%RME2 = \frac{MaxDNA_{testchemical}(\mu g/well) - DNA_{negativecontrol}(\mu g/well)}{MaxDNA_{E2}(\mu g/well) - DNA_{negativecontrol}(\mu g/well)} \times 100\%$$

If a test chemical has a positive response, but an EC50 can not be calculated because all criteria are not met, then the estrogenicity of the test chemical is simply characterized as EA positive or by its %RME2. ***Note that EC50s as calculated using Equation 1 give a measure of binding affinity (but not response amplitude), whereas %RME2s as calculated using Equation 2 give a measure of response amplitude (but not binding affinity).***

The average of three or four replicates for each concentration are plotted as relative EA (% E2) with E2 = 100% and negative control (extract solution) = 0%. The standard deviations for the replicates are so small that they fall within space taken up by plots of averages of data for each concentration. The EC50 of E2 (50%E2) in this assay is historically 10^{-13}M - 10^{-12}M depending on starting cell density/well and the maximum E2 response is attained at ~10^{-11}M.

The EA of a test chemical or extract is considered detectable if it produces cell proliferation that is greater than 15% of the maximum response to E2 (aka > 15%RME2), which is greater than three standard deviations from the historic control baseline response (about 10^{-15}M), i.e. a rather conservative measure of EA detectability. MCF-7 cell proliferation to 10^{-11}M E2 is typically used as the positive reference control in assays of extracts to insure that E2 is indeed at maximum value. Note that for assays of extracts (**Figure 1 D-F** in main paper), we typically dilute 10^{-9}M E2 100x to obtain 10^{-11}M E2. Note that negative %E2 values can occur at concentrations at which a test chemical or extract induces less proliferation than the standard negative control; %RME2 values greater than 100% can occur when the chemical or extract induces more cell proliferation than the maximum produced by E2.

If a test chemical or extract does not induce MCF-7 cell proliferation or the stimulated cell proliferation cannot be inhibited by ICI 182,780, and all positive controls (E2 and/or EA- positive plastic resin control) show detectable EA, then the test chemical is said to have no detectable EA.

EA Assay Acceptance Criteria:

1) The negative control is the vehicle control. The vehicle control must not stimulate cell growth.
2) E2 must stimulate cell growth by at least 3 fold.
3) The EC50 of E2 must be within 2.5 SD of the historical mean established by CCi and have an R^2 (coefficient of determination) value ≥ 0.9 calculated by Hill kinetics.
4) The EC50 of a test chemical cannot be calculated unless the dose-response curve of a test chemical meet the following criteria: at least one data point on each EC50 plot of E2 control must be 10% - 50% of the maximum response and at least one data point must be 50% - 100% of the maximum response. At least two

data points must be between 10% of the minimum (vehicle control) response, and an R^2 (coefficient of determination) value ≥ 0.8 calculated by Hill kinetics.

5) A weak plastic positive control must stimulate cell growth by at least 2 fold.
6) The stimulation of cell proliferation must be inhibited by ICI 182,780 at 10^{-7}M - 10^{-8}M.

Calculation of anti-EA: As per ICCVAM recommendations, our robotic anti-EA assay uses $2x10^{-12}$M E2 to obtain an EC80 level of E2-stimulated MCF-7 cell proliferation (DNA_{E2}). [Our historical data show that the EC80 of E2 is about $2x10^{-12}$M in our robotic EA assay] The data are plotted as relative anti-EA with ICI = 100% and negative control = 0%. If there is no cellular toxicity at higher concentrations, the anti-EA of a test chemical or extract can then be expressed as the IC50 concentration (in M) needed to reduce the E2-stimulated cell proliferation by 50% (IC50). When an IC50 cannot be calculated (dose-response curves do not meet the anti-EA acceptance criteria described below), the (normalized) anti-EA of a test chemical or extracts from plastics is expressed as percentage of normalized anti-EA of ICI 182,780 (ICI, positive control) as calculated by **Equation 3**:

Equation 3

$$\text{Normalized \%ICI} = \frac{DNA_{E2}(\mu g/well) - DNA_{testchemical}(\mu g/well)}{DNA_{E2}(\mu g/well) - DNA_{ICI}(\mu g/well)} \times 100\%$$

Plots of a single test run showing IC50 data for test or control chemicals are calculated as the mean of at least three replicates for each concentration. If a test chemical or extract does not inhibit E2-induced MCF-7 cell proliferation, and all positive controls (ICI, and/or dibenzoanthracene) show detectable anti-EA then the test chemical or extract is said to have no detectable anti-EA. If inhibition of E2-stimlated MCF-7 cell proliferation by a test chemical cannot be reversed by the presence of higher concentration of E2, then the test chemical or extract is considered to have no detectable anti-EA. Such inhibition is almost-certainly caused by cytotoxicity that is examined in a separate assay.

Anti-EA Assay Acceptance Criteria:

1) The negative control is the vehicle control. The vehicle control must not inhibit $2x10^{-12}$M E2-stimulated cell growth.
2) E2 must stimulate cell growth by at least 3 fold.
3) The IC50 of ICI 182,780 calculated by measuring the reduction in cell proliferation produced by $2x10^{-12}$M E2 by ICI in dilutions of 10^{-7}M to 10^{-13}M. The IC50 of ICI 182,780 must be within 2.5 SD of our historical mean established and have an R^2 (coefficient of determination) value ≥ 0.9 calculated by Hill kinetics. The reduction of E2-stimulated cell growth by ICI must be ≥ 3 fold
4) The IC50 of this test chemical can not be calculated unless the dose-response curve of a test chemical meets the following criteria: At least one data point on each IC50 plot must be 10% - 50% of the maximum response and at least one data

point must be 50%- 100% of the maximum response; At least two data points must be between the minimum (vehicle control) response and the maximum response (10^{-7}M ICI), and an R^2 (coefficient of determination) value ≥ 0.8 calculated by Hill kinetics

5) A test chemical or extract is considered to be anti-EA positive without a calculated IC50 if its dose-response curve does not meet the above criteria, but its reduction of cell proliferation is reversed by 100 - 1000 fold increases in [E2].

MATERIALS AND PROCESSES IN PLASTICS PRODUCTION

Though outwardly simple, a plastic product, such as a recently-purchased baby bottle, uses many chemicals, chemical reactions, manufacturing processes, and decorating techniques. Plastic parts are typically made from resins using manufacturing processes (**Supplemental Material, Figure 1**) that collectively incorporate into the plastic part a great variety of chemicals, many of which might be expected to exhibit EA because they have physical-chemical properties, often an insufficiently-hindered phenol group, that enable them to bind to ERs (**Supplemental Material, Table 1**). **Supplemental Material, Figure 1** is a simplified schematic of the production of only one component part of a plastic product, e.g., the bottle component of a contemporary baby bottle. Other components, such as the cap, gasket, and nipple have their own resin formulations and processing techniques, and hence increase the number of chemicals and processes used to make the final product. That is, each plastic part is usually a rather unique combination of 5-30 chemicals. A more complex plastic product (e.g., a baby bottle) often has 30 - 100 or more different chemicals (some unintended, such as catalyst residues), all of which can typically leach from the product -- any of which might have EA** -- and none of which are typically revealed to the public because the chemical composition of the product is proprietary.

There are two principal classes of plastics, thermoplastics and thermosets. Thermoplastics are polymers which can be melted and solidified many times while thermosets are polymers whose final molded form is irreversible. Thermoplastics are the most prevalent plastics in the U.S. for food and beverage packaging due to their cost, performance, and ease of processing. Some examples of thermoplastic resin types are: COC, COP, Co-PET, HDPE, LDPE, PC, PE, PES, PET, PETG, PLA, PP, and PPCO (see main paper for definition of each abbreviation). Thermoset polymers include liquid silicone rubbers (used for bottle nipples), phenolics, epoxies, and polyurethanes. Baby bottles usually have component parts from each class.

Supplemental Material, Figure 1: Production of a typical plastic part

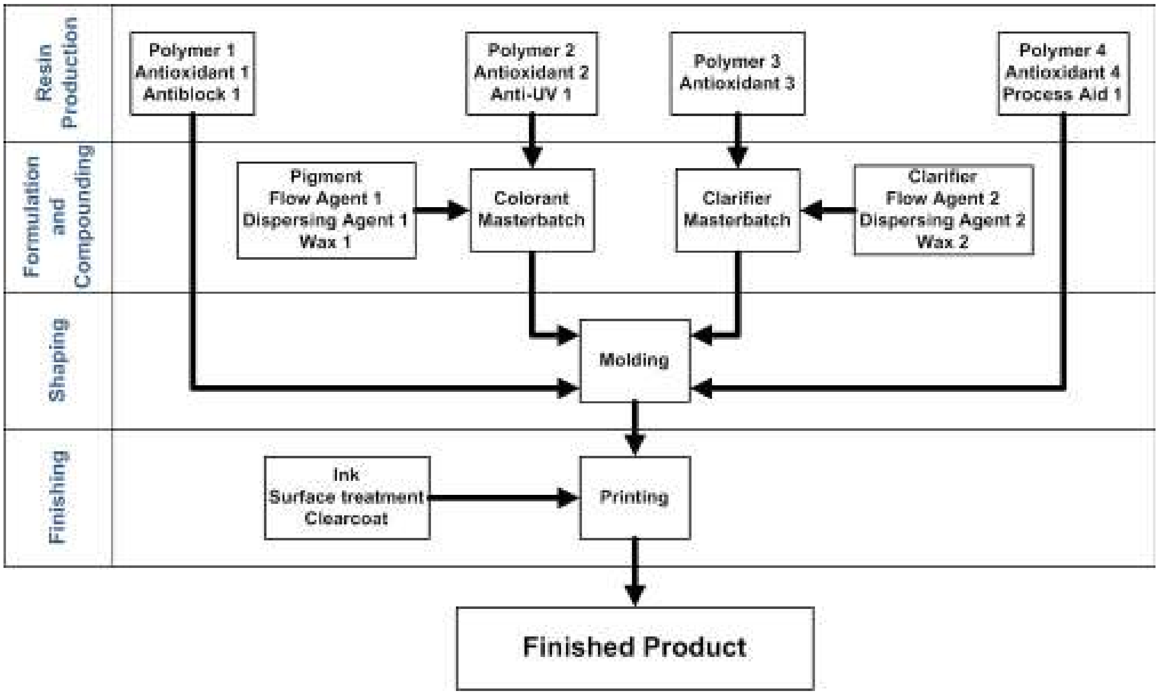

Legend. Diagram of steps and materials used to make a typical thermoplastic part starting with raw materials. A thermoplastic resin producer will begin with various monomers, link them together into long chains ("polymers"), which are subsequently mixed with small quantities of various additives (antioxidants, plasticizers, clarifiers, etc) and melted, mixed, extruded, and pelletized to form a resin. Resins are either used as-is or, more commonly, mixed with other resins, additives, colorants, lubricants, and/or extenders to form plastic compound resins (e.g., polymer blends and pre-colored polymers). Next, a compounder will combine additives that impart color, clarity, impact resistance, flexibility, and other desired final product qualities with dispersing agents and a carrier resin in specialized extruders to form a "masterbatch" material, which is essentially a concentrated form of the additives, suitable for adding to base resins. An end processor will then typically blend one or more base resins or compounds with one or more masterbatches to engineer the final product's look, feel, and performance before melting the blend for shaping. The final shaping or conversion usually involves molding and/or extrusion processes that expose the blend to heat and shear stresses. Once formed, the product is then decorated by printing and overcoating, which add polymer inks, pigments, and other additives to the chemical mix whose composition is proprietary and almost never revealed.

Thermoplastics used for most bottle component parts are made by polymerizing a specific monomer or monomers in the presence of catalysts into a high molecular weight chain known as a thermoplastic **polymer.** The resulting polymer is mixed with small quantities of various additives (antioxidants, plasticizers, clarifiers, etc) and melted, mixed, extruded, and pelletized to form a base thermoplastic **resin**. Base resins are either used as-is (e.g., BPA-based PC non-BPA-based PPCO, and non-BPA-based PPHO) or, more commonly, mixed with other resins, additives, colorants, and/or extenders to form plastic **compounds (**e.g., polymer blends and pre-colored polymers). Plastic products are then made by conversion processes (e.g., molding) using one or more plastic compounds or resins to form a finished plastic part that can be subjected to finishing processes that may utilize inks, adhesives, etc. (all of which often use chemicals having EA or anti-EA), to make a finished product.

Almost all of the 5-30 chemicals that are used to make a component plastic part can leach from any plastic product because polymerization is almost always incomplete (leaving residual unincorporated monomers) and/or because most additives (e.g., antioxidants) are not chemically part of the polymeric structure (Begley et al. 2005; De Meulenaer and Huyghebaert 2004; Le et al. 2008). Unless the selection of chemicals is carefully controlled, some of those chemicals will almost certainly have EA** -- especially since many phenolics are typically used in all steps of manufacturing and decorating processes. An EA-free resin may end up in an EA**-containing product because chemicals in various finishing or marking materials had EA**.

Even when using all materials that initially test EA-free**, the stresses of manufacturing can change chemical structures or create chemical reactions to convert an EA-free** chemical into a chemical having EA**. Furthermore, after the product is purchased and put to its intended use, multiple common-use stresses such as UV light, microwave radiation, moist heat, or freezing can also convert EA-free** polymers or additives into chemicals exhibiting EA**. Accounting for each and all such factors may appear obvious or mundane, but are essential to producing EA-free** parts and products. Only recently have a few of these factors been explicitly considered individually, such as avoiding the use of individual chemicals having easily-detectable EA** (e.g., BPA, some phthalates). These factors have not been considered in aggregate by any scientific publication, government agency or commercial entity to date of which we are aware.

Supplemental Material, Table 1 describes some quantitative structure-activity relationships (QSAR) of chemicals that help us predict whether a chemical will bind to mammalian ERs.

Supplemental Material, Table 1: Physical-chemical properties of chemicals that affect binding to ERs. Based in part on Fang et al. 2003.

**1. The molecular weight**. The MW of phenol, 94 daltons, is assumed to be the lowest limit for a xenobiotic to bind to an ER, whereas 1000 daltons is assumed to be the upper limit of ER binding. Most chemicals having MW's >= 600 Daltons will not bind to the ERs. However, the degradation products of many chemicals exposed to stresses (heat, moisture, UV) experienced by plastic in normal use may bind to ERs. This result is often seen for chemicals having phenolic groups bound to linear carbon chains.
**2. A ring structure.** If a chemical contains no ring structure, it is exceedingly unlikely to be an ER ligand. A more rigid ring (e.g., benzene, triazine) structure favors ER binding. If a chemical has a non-aromatic ring structure, it is unlikely to be an ER ligand, assuming that it does not contain an O, S, N or other H-bonding atom. However, a non-aromatic chemical may transform into an aromatic structure in the presence of heat, catalysts, exposure to light, or other stresses**.**
**3. A phenolic ring**. The presence of a single phenolic (or hydroxyl triazine) ring is much more significant than any other structural or physical-chemical feature. The phenolic 3-OH group acts primarily as H-bond donor, although it can also act as an acceptor. The H-donor ability of the 3-OH group is especially affected by the nature of immediately adjacent ortho or para groups. The H-bond donor ability for several ortho- or para-substituted phenols show the trend: phenol > 2-methylphenol = 2-t-butylphenol > 2,6-dimethylphenol > 2,6-di-t-butylphenol, in which 2,6-di-t-butylphenol is not an H-bond donor, consistent with the lack of binding activity observed for 4,4'-methylenebis(2,6-di-*t*-butylphenol). A chemical with a phenolic structure is likely to bind to ER, but the degree of potency is dependent on the presence of key structural features described in 4-7 below. If a xenobiotic has a benzene ring without an -OH group, it can still bind to the ER, although its binding potential is then heavily dependent on the presence of key structural features described in 6-7 below. Furthermore, OH, Cl, and other groups that have appropriate characteristics described in 6-7 below are easily added to hexane, hexene, or benzene rings under stress conditions experienced by many plastic products in normal use.
**4. An H-bond donor mimicking the 17β-OH**.
**5. The distance between the oxygen atoms at 3-OH and 17β-OH**. The spacing of two -OH groups at either end of a planar, and primarily hydrophobic, chemical is critical for ER binding. Chemicals containing only one phenolic group are likely to have weak to medium affinity for the ER. Most strong to medium ER ligands contain two -OH groups with an O-O distance ranging from 9.7 -12.3 Å.
6. **Steric hydrophobic centers mimicking 7α and 11β steric configuration of 17β-estradiol.** The precise steric size and orientation of the hydrophobic groups is as important as a 17β-OH. Chemicals containing a phenolic ring separated from another benzene ring with 0-3 bridge atoms will most likely be an ER ligand. The volume of the ER ligand-binding pocket (450 $Å^3$) is about twice as that of 17β-estradiol (245 $Å^3$). The length and breadth of the 17β-estradiol skeleton are well matched by the geometry of the ligand binding pocket, but there are large unoccupied cavities at the 7α and 11β -positions of 17β-estradiol. The positions of these cavities allow steric groups of certain sizes to fit, and are of great importance for various xenoestrogens, some of which do not have a benzene ring or OH groups.
**7. The hydrophobicity of the entire molecule**. The ligand-binding pocket, determined by X-ray crystallography of ERs, has a 3D "cross"-like shape with center and vertical ends mainly hydrophobic, and polar groups located at opposite ends of the horizontal cavity. When a direct comparison can be made in cases when properties 1-6 above are held constant, then increased hydrophobicity usually produces greater ER affinity. Xenoestrogens with groups lacking the most effective O-O distance require greater hydrophobicity to attain the same binding affinity exhibited by 17β-estradiol or xenoestrogens with 3α and 17β –OH groups.

Supplemental Material, Tables 2A-C summarize data on the percent of commercially available food-containing plastic products (**%D**) that have detectable EA (15%RME2) **not yet exposed to common-use stresses**. The products are grouped by whether the plastic container had contents (removed before analyzing the plastic container), and whether the container had no contents at the time of purchase. The products are also grouped by different categories of:

1) Plastic resins used to manufacture the product. [As discussed above, the manufacturing process can easily add EA-containing chemicals to an EA-free resin.].
2) Type of plastic product [bags, baby bottles, water bottles, etc].
3) Type of retail outlet where the product was purchased [large retail chains not specializing in organic products vs. large retail chains specializing in organic products advertised for their health benefits].

One advantage of this particular study is that it realistically represents variability in release of chemicals having EA expected for purchase of commercially available plastic products. As described in the main paper, on average whether or not a product had contents before purchasing does not on average affect whether or not the product had detectable EA. That does not mean that the contents do not affect the release of chemicals having detectable EA in individual products. For example, on average the contents may decrease the ability to detect chemicals having EA from some products (perhaps by extracting them). Conversely, chemicals having EA in the contents can be absorbed by the polymer of the container -- perhaps because the contents contained a common food antioxidant like BHA or BHT having EA. Most of the contents of such a bottle can be quickly extracted. The BHA or BHT absorbed in the walls of the container will be extracted much more slowly.

Table 1 in the main paper and Supplemental Material, Table 2 summarize the detailed data presented in Supplemental Material, Table 5. Because of its length (over 20 pages), we have placed **Supplemental Material, Table 5** at the end of this Supplemental Material. Note that each data point in **Supplemental Material, Table 5** is the %RME2 derived from a dose-response curve for an extract of each of 455 plastic items, some of which (117) are analyzed using two or more protocols. **Table 1 in the main paper** summarizes data for all these 455 unstressed plastic products that had one or more assays whether or not the container ever had contents. **Table 2 in the main paper** summarizes the data for 102 of these unstressed plastic items that had two assays whether or not the item ever had contents. Supplemental Material, Tables 2A-C summarize the data for the 455 unstressed plastic products for each assay type according to whether the product had contents or did not have contents at the vtime of purchase.

Supplemental Material, Tables 2A-C shows that that most (71%) unstressed plastic items released chemicals having reliably-detectable EA in one or more extraction protocols, independent of whether or not the item had contents. This conclusion holds for the average numbers of items having detectable EA for different resin types, product types, or retailer types. There is no significant difference ($p > 0.05$) in the percentage of items having EA that had contents (69%, n=296) *versus* no contents (76%, n=160). The percentage of samples having EA or the mean %RME2 in individual subcategories of items also did not vary in any consistent manner between items with contents *versus*

items without contents. **Most importantly, items without contents in all categories exhibited detectable EA in at least one protocol,** including 78% of items made from HDPE (n=18), 57% from PP (n=14), and 100% from PET (n=6). Given all these results, we presented the data for all items tested in **Tables 1-2 in the main paper** as representative for plastic items with or without contents. As noted in the main paper, this lack of significant difference in average percentage having detectable EA between plastic items with and without contents does not imply that the contents do not affect the total EA or specific chemicals having EA released by individual plastic items.

Supplemental Material, Tables 2A-C also shows that the leaching of a chemical having EA was significantly ($p < 0.01$) more likely to be detected if both polar (saline) and non-polar (EtOH) solvents were used (61%) than if only one solvent were used (15% or 31%) independent of whether or not the container had contents. This conclusion holds for products made from different resins, different product types, and products sold by different retailers.

Supplemental Material, Tables 2A-C: Number (N) and percent of unstressed plastic items with or without contents having EA (%D)

Supplemental Material, Table 2A: Standard EtOH Extraction Protocol

	Standard EtOH				
	N	With Content		Without Content	
Resin Type		N	%D	N	%D
**HDPE**	13	4	25	9	89
**PP**	23	15	67	8	25
**PET**	30	26	35	4	75
**Polystyrene**	13	11	64	2	50
**PLA**	10	4	75	6	67
**PC**	1	--	--	1	0
Product Type					
**Flexible Packaging**	82	82	66	--	--
**Food Wrap**	9	--	--	9	100
**Rigid Packaging**	57	57	56	--	--
**Baby Bottle Components**	13	--	--	13	69
**Deli Containers**	11	6	67	5	0
**Plastic Bags**	33	2	100	31	97
Product Retailer					
**Large Retailer 1**	31	29	79	2	100
**Large Retailer 2**	4	3	33	1	100
**Large Retailer 3**	18	16	81	2	100
**Large Retailer 4**	37	29	38	8	100
**Large Retailer 5**	20	19	47	1	100
**Organic Retailer 1**	28	28	71	--	--
**Organic Retailer 2**	33	23	83	10	100

Items tested by standard EtOH assay and at least one other assay				
N	With Content		Without Content	
	N	%D	N	%D
308	200	67	108	70

Items tested only by standard EtOH assay				
N	With Content		Without Content	
	N	%D	N	%D
308	200	67	108	70

Supplemental Material, Table 2B: Concentrated EtOH Extraction Protocol

	Concentrated EtOH				
	N	With Content		Without Content	
Resin Type		N	%D	N	%D
**HDPE**	11	3	67	8	50
**PP**	6	2	50	4	25
**PET**	17	17	94	--	--
**Polystyrene**	--	--	--	--	--
**PLA**	1	1	100	--	--
**PC**	1	--	--	1	100
Product Type					
**Flexible Packaging**	6	6	33	--	--
**Food Wrap**	--	--	--	--	--
**Rigid Packaging**	18	12	67	6	67
**Baby Bottle Components**	--	--	--	--	--
**Deli Containers**	--	--	--	--	--
**Plastic Bags**	1	--	--	1	100
Product Retailer					
**Large Retailer 1**	2	2	100	--	--
**Large Retailer 2**	4	1	0	3	0
**Large Retailer 3**	2	2	100	--	--
**Large Retailer 4**	--	--	--	--	--
**Large Retailer 5**	3	3	100	--	--
**Organic Retailer 1**	5	5	60	--	--
**Organic Retailer 2**	1	--	--	1	100

Items tested by concentrated EtOH assay and at least one other assay				
N	With Content		Without Content	
	N	%D	N	%D
51	37	73	14	71

Items tested only by concentrated EtOH assay				
N	With Content		Without Content	
	N	%D	N	%D
10	9	56	1	0

Supplemental Material, Table 2C: Standard Saline Extraction Protocol

	Saline				
	N	With Content		Without Content	
Resin Type		N	%D	N	%D
HDPE	18	5	60	13	54
PP	16	6	83	10	80
PET	34	29	72	5	100
Polystyrene	16	5	40	11	36
PLA	8	2	100	6	100
PC	2	--	--	2	100
Product Type					
Flexible Packaging	35	35	74	--	--
Food Wrap	9	--	--	9	78
Rigid Packaging	31	25	48	6	33
Baby Bottle Components	16	--	--	16	94
Deli Containers	7	5	60	2	100
Plastic Bags	23	--	--	23	96
Product Retailer					
Large Retailer 1	4	4	75	--	--
Large Retailer 2	50	26	85	14	36
Large Retailer 3	7	6	33	1	0
Large Retailer 4	--	--	--	--	--
Large Retailer 5	4	4	100	--	--
Organic Retailer 1	5	5	80	--	--
Organic Retailer 2	10	1	100	9	78

Items tested by saline assay and at least one other assay				
N	With Content		Without Content	
	N	%D	N	%D
214	101	62	113	75

Items tested only by saline assay				
N	With Content		Without Content	
	N	%D	N	%D
116	76	66	40	68

Monomers and Additives in Plastics

Monomers: Figure 3 in the main paper shows the EA** of monomers for the most common thermoplastics. The pure ("barefoot") resins of most non-transparent flexible thermoplastics do not exhibit EA**, in large part because they do not have benzene or phenolic rings. In contrast, most HC thermoplastics have EA**. For a polymer to be HC it must have limited or no ability to crystallize and it must have a high glass transition temperature (T_g) well above room temperature. To have limited or no ability to crystallize, polymers typically have limited backbone symmetry, limited flexibility in the main chain segments and lack intermolecular polar or hydrogen bonds. To have a high T_g, polymers typically have limited backbone symmetry, short main chain segments, bulky side groups, high intermolecular forces, strong hydrogen bonding and high molecular weight. Since polymers made from phenolic-based monomers usually have an amorphous structure and a high T_g, their chemistry has been well studied and utilized to make HC polymers. However, as illustrated in **Figure 3 in the main paper**, COP/COC HC polymers produced from cyclic olefin monomers can also have a crystalline structure, high T_g and can be both EA-free** and non-toxic. Their chemistry is less well explored than HDPE, PP, PET, PS, PLA, and PC.

Antioxidant Additives: Additives are chemicals that are introduced in small quantities to enhance a plastic's physical properties (e.g., plasticizers and slip agents), visual characteristics (e.g., clarifiers and colorants), or processability (e.g., antioxidants). Antioxidant additives are almost always a required component of almost all polymer resins, while other additives (e.g., colorants and slip agents) are optionally introduced during the conversion process. Antioxidants are the most critical additives because they prevent or minimize plastic degradation due to oxidation of a polymer to form carboxyl groups that break molecular chains (chain scission), which leads to discoloration, loss of surface gloss, surface cracking, and lowering of tensile strength (Schwarzenbach et al. 2001). The oldest and most common antioxidants deemed suitable for food contact belong to a chemical class known as hindered phenols. Organophosphites are a class of antioxidant commonly used with hindered phenols to provide synergistic oxidation protection, as well as whitening. Most organophosphites in their unaltered state should not bind to ERs. However, most organophosphites are also hydrolytically unstable and produce phenols when exposed to water (Schwarzenbach et al. 2001) and, therefore, usually cannot be used to produce EA-free** plastics. **Supplemental Material, Table 3** shows the %RME2 of four unstressed antioxidants commonly used in plastic resins.

Supplemental Material, Table 3: EA of some common antioxidants

	Extract %RME2	
Antioxidant	EtOH	Saline
Phos AOX 1	0	-3
HP AOX1	**104**	2
HP AOX2	5	-2
Ph AOX 1	**60**	nt

Legend. Results (%RME2) of EA assays of four selected unstressed antioxidants; Phos = phosphate; HP = hindered phenolic; Ph = bisphenol; nt = not tested. Boldfaced numbers are %RME2 values well above detection threshold (15%RME2).

Colorant Additives: The addition of colorants to a plastic formulation during the conversion process allows a firm to differentiate its product not only by its shape and design, but also by its color. Color can also provide the plastic product with additional UV protection. Supplemental Material, Table 4 shows that many common colorants test positive for EA** (> 15%RME2).

There are two basic types of colorants used in plastic products, inorganic and organic. QSAR analyses based on **Supplemental Material, Table 1** predict, and our testing data confirm, that inorganic colorants typically do not exhibit EA**. The widespread use of inorganic colorants in plastic products is limited due to issues of availability, processability, or FDA-published toxicities. Organic colorants are more readily available, but many exhibit EA due to cyclic aromatic moieties. We have tested many colorants, and have found more than 40 inorganic and organic colorants that do not have EA** or FDA-published toxicities. Inorganic colorants are generally supplied as concentrated compounds, where a finely powdered inorganic pigment, usually a metal oxide, is dispersed in a plastic carrier. The carriers generally are a mix of low molecular weight polymers, waxes, and dispersing agents, all of which to be potentially usable to make an EA-free product must be tested to be EA-free (<15%RME2) using our MCF-7 assay.

Other Additives: Although not as critical to plastic manufacturing processes as antioxidants or colorants, other additives are utilized to improve the physical properties, optical properties (e.g. clarifiers) and marketability of a finished plastic product. A converter may use a purge compound to clean out the equipment and allow the converter to more efficiently transition between materials and products. However, most purge compounds used by converters are based on polystyrene and have EA. Converters may also use items such as mold release agents and mold cleaners. We have identified purge and mold release agents that have or do not have detectable EA.

In summary, even when using an EA-free resin, the resulting finished product can exhibit EA** due to additives used during conversion and/or finishing processes. Hence, EA-free** additives must be used in *all* stages of the manufacturing process**. As discussed above, even when EA-free** monomers and additives are used, the final plastic item must still be tested for release of chemicals having EA** because manufacturing processes or common-use stresses can convert EA-free** chemicals to chemicals having EA**.**

Supplemental Material, Table 4: EA of some chemicals used during conversion and/or finishing processes

Chemical	Classification	Detectable EA (>15%RME2)
GM	Additive-Antistat/Mold Release	Not Detected
ANA-21	Additive-Clarifier	Not Detected
M88	Additive-Clarifier	Not Detected
M00	Additive-Clarifier	Not Detected
Blue Color Concentrate	Additive-Colorant	Detected
Dark Red Color Concentrate	Additive-Colorant	Not Detected
Bronze Color Concentrate	Additive-Colorant	Not Detected
Turquoise Color Concentrate	Additive-Colorant	Detected
Gray Color Concentrate	Additive-Colorant	Not Detected
Green Color Concentrate	Additive-Colorant	Detected
Inorganic Blue Color Concentrate	Additive-Colorant	Not Detected
Light Turquoise Color Concentrate	Additive-Colorant	Detected
Light Blue Color Concentrate	Additive-Colorant	Detected
Light Brown Color Concentrate	Additive-Colorant	Detected
Light Green Color Concentrate	Additive-Colorant	Not Detected
Medium Turquoise Color Concentrate	Additive-Colorant	Detected
Red Color Concentrate	Additive-Colorant	Not Detected
Teal Color Concentrate	Additive-Colorant	Not Detected
White Color Concentrate	Additive-Colorant	Not Detected
Yellow Color Concentrate	Additive-Colorant	Not Detected
Migrating Slip Agent 1	Additive-Slip Agent	Not Detected
Migrating Slip Agent 2	Additive-Slip Agent	Not Detected
Mold Cleaner	Processing Aid-Mold Cleaner	Detected
EI Mold Release	Processing Aid-Mold Release	Not Detected
Food Approved Mold Release 1	Processing Aid-Mold Release	Detected
Food Approved Mold Release 2	Processing Aid-Mold Release	Not Detected
U Release	Processing Aid-Mold Release	Not Detected
VL Mold Release	Processing Aid-Mold Release	Not Detected
Purge Compound 1	Processing Aid-Purging Compound	Not Detected
Purge Compound 2	Processing Aid-Purging Compound	Not Detected
Purge Compound 3	Processing Aid-Purging Compound	Not Detected

References

Begley T, Castle L, Feigenbaum A, Franz R, Hinrichs K, Lickly T, et al. 2005. Evaluation of migration models that might be used in support of regulations for food-contact plastics. Food Addit Contam 22:73-90.

Burton K. 1956. A study of the conditions and mechanism of the diphenylamine reaction for the calorimetric estimation of deoxyribonucleic acid. Biochem J 62:315-323.

De Meulenaer B, Huyghebaert A. 2004. Packaging and other food contact material residues. In: Handbook of Food Analysis, Vol. 2, 2nd Ed. (Nollet LML, ed) New York:Marcel Dekker, 1297-1330.

Fang H, Tong W, Welsh WJ, Sheehan DM. 2003. QSAR Models in receptor-mediated effects: the nuclear receptor superfamily. J Mol Struct: THEOCHEM 622:113-125.

He B, Rhodes-Brower S, Miller MR, Munson AE, Germolec DR, Walker VR, et al. 2003. Oxtamethylcyclotetrasiloxane exhibits estrogenic activity in mice via ERα. Toxicol Appl Pharmacol 192:254-261.

Le HH, Carlson EM, Chua JP, Belcher SM. 2008. Bisphenol A released from polycarbonate drinking bottles mimics the neurotoxic actions of estrogen in developing cerebellar neurons. Toxicol Lett 176:149–156.

Leusch FDL, de Jager C, Levi Y, Lim R, Puijker L, Sacher F, et al. 2010. Comparison of five in vitro bioassays to measure estrogenic activity in environmental waters. Environ Sci Technol 44:3853-3860.

Natarajan N, Shambaugh GE, III, Elseth KM, Haines GK, Radosevich JA. 1994. Adaptation of the diphenylamine (DPA) assay to a 96-well plate tissue culture format and comparison with the MTT assay. BioTechniques 17:166-171.

Schwarzenbach K, Gilg B, Müller D, Knobloch G, Pauquet J-R, Rota-Graziosi P, et al. 2001. Antioxidants. In: Plastics Additives Handbook, 5th Ed (Zweifel H, ed). Munich:Hanser, 1-136.

Soto AM, Sonnenschein C, Chung, KL, Fernandez MF, Olea N, Serrano FO. 1995. The E-SCREEN assay as a tool to identify estrogens: an update on estrogenic environmental pollutants. Environ Health Perspect 103:113-122.

Taylor JA, Grady LH, Engler KS and Welshons WV. 1995. Relationship of growth stimulated by lithium, estradiol and EGF to phospholipase C activity in MCF-7 human breast cancer cells. Breast Cancer Res Treat 34:265-277.

Summary of Supplemental Materials, Tables 5A-T

Supplemental Material, Tables 5A-S: %RME2 for individual samples with and without contents of a given classification (resin type, functional use, class of retailer) and the mean %RME2 ± SD for all samples having EA (>15%RME2) for each extraction protocol for that given classification.

Supplemental Material, Table 5T: mean %RME2 ± SD for all samples having EA (>15%RME2) for each extraction protocol for all classification with contents, without contents, or with or without contents.

For each table in **Supplemental Material, Tables 5A-S**, sample numbers (first column: n_1 - n_x) are assigned in order of increasing EA (second column: %RME2 values), and the last column gives whether the item had contents at the time of purchase (y = yes, n = no). The same sample number (1, 2, 3, etc.) for different extraction protocols usually does not refer to the same item. The %RME2 for each sample is calculated (**Equation 2)** from its %E2 dilution curve relative to E2 (**Equation 1**) and confirmed by ICI suppression (see **Fig. 1D-F in main paper**). The mean and standard deviation (SD) is calculated for %RME2 values for all samples in each category having detectable %RME2 > 15%. N= number of samples for each calculation; $Mean_D$ = mean of samples having detectable EA; SD_D = SD of all samples having detectable EA; N_D = N number of samples having detectable EA.

The first set of $Mean_D$ ± SD_D and N_D values given for each extraction protocol for each subtable (e.g., 5A, 5B, etc) are for all items having detectable EA with or without contents, the next set of $Mean_D$ ± SD_D and N_D values are for items having detectable EA purchased with contents and the last set of $Mean_D$ ± SD_D and N_D values are for items having detectable EA purchased without contents.

Supplemental Material, Table 5A: %RME2 values for individual samples manufactured using HDPE, and mean %RME2 ± SD for all samples for each extraction protocol

%RME2 with and without content

$Mean_D$	66
SD_D	25
N_D	9

%RME2 with and without content

$Mean_D$	56
SD_D	20
N_D	7

%RME2 with and without content

$Mean_D$	33
SD_D	16
N_D	10

%RME2 with content

$Mean_D$	58
SD_D	--
N_D	1

%RME2 with content

$Mean_D$	62
SD_D	6
N_D	2

%RME2 with content

$Mean_D$	44
SD_D	23
N_D	2

%RME2 without content

$Mean_D$	67
SD_D	27
N_D	8

%RME2 without content

$Mean_D$	54
SD_D	24
N_D	5

%RME2 without content

$Mean_D$	31
SD_D	15
N_D	8

Sample # HDPE	%RME2 Std EtOH	Content
1	0	y
2	3	n
3	5	y
4	15	y
5	19	n
6	50	n
7	54	n
8	58	y
9	62	n
10	74	n
11	84	n
12	91	n
13	102	n

Sample # HDPE	%RME2 Conc EtOH	Content
1	-3	n
2	1	n
3	3	n
4	15	y
5	16	n
6	43	n
7	58	y
8	66	y
9	68	n
10	69	n
11	73	n

Sample # HDPE	%RME2 Saline	Content
1	-4	n
2	2	n
3	3	n
4	5	y
5	6	n
6	10	y
7	10	n
8	11	n
9	16	n
10	18	n
11	20	n
12	24	n
13	27	y
14	30	n
15	32	n
16	47	n
17	58	n
18	60	y

Supplemental Material, Table 5B: %RME2 values for individual samples manufactured using PP and mean %RME2 ± SD for all samples for each extraction protocol

%RME2 with and without content

$Mean_D$	42
SD_D	23
N_D	14

%RME2 with content

$Mean_D$	42
SD_D	24
N_D	11

%RME2 without content

$Mean_D$	41
SD_D	25
N_D	3

Sample # PP	%RME2 Std EtOH	Content
1	-3	**n**
2	-2	**y**
3	-1	**n**
4	-1	**n**
5	0	**y**
6	1	**y**
7	8	**y**
8	8	**n**
9	10	**n**
10	23	**y**
11	25	**n**
12	28	**y**
13	28	**n**
14	29	**y**
15	30	**y**
16	34	**y**
17	34	**y**
18	40	**y**
19	40	**y**
20	41	**y**
21	56	**y**
22	71	**n**
23	110	**y**

%RME2 with and without content

$Mean_D$	25
SD_D	3
N_D	4

%RME2 with content

$Mean_D$	28
SD_D	--
N_D	1

%RME2 without content

$Mean_D$	24
SD_D	3
N_D	3

Sample # PP	%RME2 Conc EtOH	Content
1	-2	**n**
2	-2	**y**
3	20	**n**
4	25	**n**
5	25	**n**
6	28	**y**

%RME2 with and without content

$Mean_D$	48
SD_D	30
N_D	12

%RME2 with content

$Mean_D$	44
SD_D	27
N_D	5

%RME2 without content

$Mean_D$	50
SD_D	34
N_D	7

Sample # PP	%RME2 Saline	Content
1	1	**y**
2	4	**n**
3	10	**n**
4	15	**n**
5	16	**n**
6	17	**n**
7	19	**y**
8	28	**y**
9	36	**n**
10	41	**y**
11	41	**n**
12	44	**y**
13	48	**n**
14	83	**n**
15	90	**y**
16	110	**n**

Supplemental Material, Table 5C: %RME2 values for individual samples manufactured using PET and mean %RME2 ± SD for all samples for each extraction protocol

%RME2 with and without content

$Mean_D$	38
SD_D	16
N_D	14

%RME2 with content

$Mean_D$	34
SD_D	14
N_D	11

%RME2 without content

$Mean_D$	55
SD_D	8
N_D	3

Sample # PET	%RME2 Std EtOH	Content
1	-1	y
2	0	y
3	0	y
4	1	y
5	2	y
6	2	y
7	4	y
8	4	y
9	5	y
10	7	y
11	7	n
12	10	y
13	11	y
14	12	y
15	12	y
16	15	y
17	17	y
18	20	y
19	22	y
20	24	y
21	25	y
22	31	y
23	36	y
24	36	y
25	45	y
26	50	n
27	52	n

%RME2 with and without content

$Mean_D$	39
SD_D	22
N_D	16

%RME2 with content

$Mean_D$	39
SD_D	22
N_D	16

%RME2 without content

$Mean_D$	--
SD_D	--
N_D	--

Sample # PET	%RME2 Conc EtOH	Content
1	10	y
2	20	y
3	22	y
4	24	y
5	24	y
6	25	y
7	25	y
8	29	y
9	31	y
10	31	y
11	33	y
12	36	y
13	45	y
14	50	y
15	62	y
16	77	y
17	99	y

%RME2 with and without content

$Mean_D$	64
SD_D	41
N_D	26

%RME2 with content

$Mean_D$	67
SD_D	43
N_D	21

%RME2 without content

$Mean_D$	50
SD_D	25
N_D	5

Sample # PET	%RME2 Saline	Content
1	-5	y
2	1	y
3	2	y
4	2	y
5	9	y
6	12	y
7	14	y
8	14	y
9	16	y
10	20	y
11	21	n
12	23	y
13	24	y
14	26	y
15	31	n
16	36	y
17	38	y
18	39	y
19	50	n
20	54	y
21	57	y
22	67	y
23	69	n
24	72	y
25	75	y
26	77	y
27	80	n

28	55	**y**
29	59	**y**
30	64	**n**

28	81	**y**
29	81	**y**
30	81	**y**
31	81	**y**
32	142	**y**
33	145	**y**
34	175	**y**

Supplemental Material, Table 5D: %RME2 values for individual samples manufactured using polystyrene, and mean %RME2 ± SD for all samples for each extraction protocol

%RME2 with and without content

$Mean_D$	61
SD_D	33
N_D	8

%RME2 with content

$Mean_D$	61
SD_D	36
N_D	7

%RME2 without content

$Mean_D$	64
SD_D	--
N_D	1

Sample # Polystyrene	%RME2 Std EtOH	Content
1	-3	y
2	2	n
3	7	y
4	7	y
5	13	y
6	19	y
7	25	y
8	48	y
9	48	y
10	64	n
11	76	y
12	89	y
13	120	y

%RME2 with and without content

$Mean_D$	51
SD_D	33
N_D	6

%RME2 with content

$Mean_D$	88
SD_D	2
N_D	2

%RME2 without content

$Mean_D$	32
SD_D	19
N_D	4

Sample # Polystyrene	%RME2 Saline	Content
1	0	y
2	3	y
3	5	y
4	7	n
5	7	n
6	7	n
7	9	n
8	9	n
9	12	n
10	14	n
11	17	n
12	20	n
13	32	n
14	58	n
15	87	y
16	90	y

Supplemental Material, Table 5E: %RME2 values for individual samples manufactured using PLA, and mean %RME2 ± SD for all samples for each extraction protocol

%RME2 with and without content

$Mean_D$	41
SD_D	11
N_D	8

%RME2 with content

$Mean_D$	35
SD_D	13
N_D	4

%RME2 without content

$Mean_D$	46
SD_D	3
N_D	4

Sample # PLA	%RME2 Std EtOH	Content
1	1	**n**
2	13	**n**
3	20	**y**
4	33	**y**
5	36	**y**
6	44	**n**
7	44	**n**
8	48	**n**
9	50	**n**
10	51	**y**

%RME2 with and without content

$Mean_D$	20
SD_D	--
N_D	1

%RME2 with content

$Mean_D$	20
SD_D	--
N_D	1

%RME2 without content

$Mean_D$	--
SD_D	--
N_D	--

Sample # PLA	%RME2 Conc EtOH	Content
1	20	**y**

%RME2 with and without content

$Mean_D$	49
SD_D	31
N_D	8

%RME2 with content

$Mean_D$	42
SD_D	12
N_D	2

%RME2 without content

$Mean_D$	51
SD_D	36
N_D	6

Sample # PLA	%RME2 Saline	Content
1	19	**n**
2	22	**n**
3	33	**y**
4	38	**n**
5	40	**n**
6	50	**y**
7	73	**n**
8	114	**n**

Supplemental Material, Table 5F: %RME2 values for individual samples manufactured using PC, and mean %RME2 ± SD for all samples for each extraction protocol

%RME2 with and without content

$Mean_D$	--
SD_D	--
N_D	--

%RME2 with content

$Mean_D$	--
SD_D	--
N_D	--

%RME2 without content

$Mean_D$	--
SD_D	--
N_D	--

Sample #	%RME2	
**PC**	**Std EtOH**	**Content**
**1**	**4**	**n**
**2**	**9**	**n**

%RME2 with and without content

$Mean_D$	91
SD_D	N/A
N_D	1

%RME2 with content

$Mean_D$	--
SD_D	--
N_D	--

%RME2 without content

$Mean_D$	91
SD_D	N/A
N_D	1

Sample #	%RME2	
**PC**	**Conc EtOH**	**Content**
1	91	**n**

%RME2 with and without content

$Mean_D$	91
SD_D	8
N_D	2

%RME2 with content

$Mean_D$	--
SD_D	--
N_D	--

%RME2 without content

$Mean_D$	91
SD_D	8
N_D	2

Sample #	%RME2	
**PC**	**Saline**	**Content**
1	85	**n**
2	97	n

Supplemental Material, Table 5G: %RME2 values for individual samples of flexible packaging, and mean %RME2 ± SD for all samples for each extraction protocol

%RME2 with and without content

$Mean_D$	49
SD_D	24
N_D	53

%RME2 with and without content

$Mean_D$	46
SD_D	17
N_D	2

%RME2 with and without content

$Mean_D$	41
SD_D	22
N_D	25

%RME2 with content

$Mean_D$	49
SD_D	24
N_D	53

%RME2 with content

$Mean_D$	46
SD_D	17
N_D	2

%RME2 with content

$Mean_D$	41
SD_D	22
N_D	25

%RME2 without content

$Mean_D$	--
SD_D	--
N_D	--

%RME2 without content

$Mean_D$	--
SD_D	--
N_D	--

%RME2 without content

$Mean_D$	--
SD_D	--
N_D	--

Sample #	%RME2	
**Flex. Pack.**	**Std EtOH**	**Content**
1	-33	y
2	-6	y
3	-1	y
4	0	y
5	1	y
6	1	y
7	1	y
8	2	y
9	2	y
10	2	y
11	2	y
12	3	y
13	3	y
14	4	y
15	5	y
16	6	y
17	7	y
18	7	y
19	8	y
20	8	y
21	9	y
22	9	y
23	9	y
24	9	y
25	9	y
26	10	y
27	10	y
28	14	y

Sample #	%RME2	
**Flex. Pack.**	**Conc EtOH**	**Content**
1	2	y
2	4	y
3	9	y
4	11	y
5	34	y
6	59	y

Sample #	%RME2	
**Flex. Pack.**	**Saline**	**Content**
1	-16	y
2	-11	y
3	-5	y
4	-4	y
5	4	y
6	4	y
7	4	y
8	5	y
9	7	y
10	15	y
11	16	y
12	18	y
13	18	y
14	18	y
15	19	y
16	19	y
17	19	y
18	22	y
19	24	y
20	26	y
21	28	y
22	35	y
23	36	y
24	43	y
25	44	y
26	47	y
27	50	y
28	55	y

29	14	y
30	16	y
31	17	y
32	19	y
33	19	y
34	21	y
35	22	y
36	23	y
37	25	y
38	26	y
39	27	y
40	27	y
41	27	y
42	28	y
43	29	y
44	29	y
45	32	y
46	33	y
47	34	y
48	34	y
49	35	y
50	35	y
51	35	y
52	38	y
53	40	y
54	40	y
55	44	y
56	45	y
57	47	y
58	48	y
59	49	y
60	50	y
61	54	y
62	55	y
63	57	y
64	59	y
65	61	y
66	62	y
67	63	y
68	63	y
69	64	y
70	64	y
71	64	y
72	68	y
73	70	y
74	71	y
75	72	y
76	76	y
77	78	y

29	56	y
30	59	y
31	64	y
32	66	y
33	71	y
34	71	y
35	94	y

78	79	**y**
79	96	**y**
80	106	**y**
81	106	**y**
82	110	**y**

Supplemental Material, Table 5H: %RME2 values for individual samples of food wrap, and mean %RME2 ± SD for all samples for each extraction protocol

%RME2 with and without content

$Mean_D$	64
SD_D	12
N_D	9

%RME2 with content

$Mean_D$	--
SD_D	--
N_D	--

%RME2 without content

$Mean_D$	64
SD_D	12
N_D	9

Sample #	%RME2	
**Food Wrap**	**Std EtOH**	**Content**
1	47	**n**
2	52	**n**
3	54	**n**
4	63	**n**
5	64	**n**
6	64	**n**
7	68	**n**
8	79	**n**
9	85	**n**

%RME2 with and without content

$Mean_D$	65
SD_D	34
N_D	7

%RME2 with content

$Mean_D$	--
SD_D	--
N_D	--

%RME2 without content

$Mean_D$	65
SD_D	34
N_D	7

Sample #	%RME2	
**Food Wrap**	**Saline**	**Content**
1	-9	**n**
2	14	**n**
3	21	**n**
4	39	**n**
5	63	**n**
6	64	**n**
7	68	**n**
8	69	**n**
9	129	**n**

Supplemental Material, Table 5I: %RME2 values for individual samples of rigid packaging, and mean %RME2 ± SD for all samples for each extraction protocol

%RME2 with and without content

$Mean_D$	43
SD_D	25
N_D	38

%RME2 with and without content

$Mean_D$	42
SD_D	22
N_D	14

%RME2 with and without content

$Mean_D$	45
SD_D	27
N_D	13

%RME2 with content

$Mean_D$	43
SD_D	25
N_D	38

%RME2 with content

$Mean_D$	36
SD_D	21
N_D	8

%RME2 with content

$Mean_D$	47
SD_D	28
N_D	11

%RME2 without content

$Mean_D$	--
SD_D	--
N_D	--

%RME2 without content

$Mean_D$	50
SD_D	24
N_D	6

%RME2 without content

$Mean_D$	33
SD_D	19
N_D	2

Sample #	%RME2	
**Rigid Pack.**	**Std EtOH**	**Content**
1	-6	**y**
2	-4	**y**
3	-3	**y**
4	-2	**y**
5	-1	**y**
6	-1	**y**
7	0	**y**
8	1	**y**
9	2	**y**
10	2	**y**
11	4	**y**
12	5	**y**
13	7	**y**
14	7	**y**
15	9	**y**
16	10	**y**
17	13	**y**
18	14	**y**
19	15	**y**
20	18	**y**
21	18	**y**
22	19	**y**
23	19	**y**
24	19	**y**
25	20	**y**
26	20	**y**
27	21	**y**
28	22	**y**

Sample #	%RME2	
**Rigid Pack.**	**Conc EtOH**	**Content**
1	-2	**y**
2	2	**y**
3	3	**y**
4	10	**y**
5	20	**y**
6	20	**y**
7	20	**n**
8	22	**y**
9	25	**n**
10	28	**y**
11	33	**y**
12	35	**y**
13	43	**n**
14	45	**y**
15	68	**n**
16	69	**n**
17	73	**n**
18	83	**y**

Sample #	%RME2	
**Rigid Pack.**	**Saline**	**Content**
1	0	**y**
2	1	**y**
3	1	**y**
4	2	**y**
5	3	**n**
6	3	**y**
7	4	**n**
8	5	**y**
9	5	**y**
10	6	**y**
11	6	**y**
12	6	**y**
13	7	**y**
14	7	**y**
15	10	**n**
16	10	**y**
17	11	**n**
18	15	**y**
19	17	**y**
20	19	**y**
21	20	**n**
22	25	**y**
23	28	**y**
24	40	**y**
25	41	**y**
26	41	**y**
27	44	**y**
28	47	**n**

29	23	y
30	25	y
31	28	y
32	29	y
33	30	y
34	33	y
35	34	y
36	34	y
37	34	y
38	35	y
39	37	y
40	37	y
41	40	y
42	40	y
43	41	y
44	45	y
45	48	y
46	48	y
47	48	y
48	49	y
49	56	y
50	59	y
51	60	y
52	76	y
53	77	y
54	83	y
55	89	y
56	110	y
57	120	y

29	87	y
30	90	y
31	90	y

Supplemental Material, Table 5J: %RME2 values for individual samples of baby bottle components, and mean %RME2 ± SD for all samples for each extraction protocol

%RME2 with and without content

$Mean_D$	49
SD_D	32
N_D	8

%RME2 with content

$Mean_D$	--
SD_D	--
N_D	--

%RME2 without content

$Mean_D$	49
SD_D	32
N_D	8

Sample #	%RME2	
**Baby Bottles Components**	**Std EtOH**	**Content**
1	-1	**n**
2	7	**n**
3	8	**n**
4	11	**n**
5	15	**n**
6	20	**n**
7	22	**n**
8	30	**n**
9	30	**n**
10	41	**n**
11	52	**n**
12	86	**n**
13	107	**n**

%RME2 with and without content

$Mean_D$	52
SD_D	31
N_D	15

%RME2 with content

$Mean_D$	--
SD_D	--
N_D	--

%RME2 without content

$Mean_D$	52
SD_D	31
N_D	15

Sample #	%RME2	
**Baby Bottles Components**	**Saline**	**Content**
1	4	**n**
2	16	**n**
3	17	**n**
4	22	**n**
5	24	**n**
6	25	**n**
7	35	**n**
8	41	**n**
9	41	**n**
10	44	**n**
11	63	**n**
12	64	**n**
13	90	**n**
14	91	**n**
15	93	**n**
16	110	**n**

Supplemental Material, Table 5K: %RME2 values for individual samples of deli containers, and mean %RME2 ± SD for all samples for each extraction protocol

%RME2 with and without content

$Mean_D$	36
SD_D	12
N_D	4

%RME2 with content

$Mean_D$	36
SD_D	12
N_D	4

%RME2 without content

$Mean_D$	--
SD_D	--
N_D	--

Sample # Deli Container	%RME2 Std EtOH	Content
1	-7	**n**
2	-3	**n**
3	-1	**n**
4	8	**y**
5	10	**y**
6	10	**n**
7	11	**n**
8	23	**y**
9	33	**y**
10	36	**y**
11	51	**y**

%RME2 with and without content

$Mean_D$	38
SD_D	9
N_D	4

%RME2 with content

$Mean_D$	41
SD_D	9
N_D	3

%RME2 without content

$Mean_D$	31
SD_D	--
N_D	1

Sample # Deli Cont.	%RME2 Saline	Content
1	2	**y**
2	9	**y**
3	15	**n**
4	31	**n**
5	33	**y**
6	39	**y**
7	50	**y**

Supplemental Material, Table 5L: %RME2 values for individual samples of plastic bags, and mean %RME2 ± SD for all samples for each extraction protocol

%RME2 with and without content

$Mean_D$	70
SD_D	29
N_D	33

%RME2 with content

$Mean_D$	98
SD_D	13
N_D	2

%RME2 without content

$Mean_D$	68
SD_D	29
N_D	31

Sample # Plastic Bags	%RME2 Std EtOH	Content
1	16	**n**
2	18	**n**
3	26	**n**
4	28	**n**
5	31	**n**
6	32	**n**
7	38	**n**
8	46	**n**
9	50	**n**
10	54	**n**
11	54	**n**
12	60	**n**
13	62	**n**
14	64	**n**
15	67	**n**
16	72	**n**
17	74	**n**
18	78	**n**
19	82	**n**
20	84	**n**
21	84	**n**
22	85	**n**
23	86	**n**
24	86	**n**
25	87	**n**
26	88	**y**
27	91	**n**
28	96	**n**

%RME2 with and without content

$Mean_D$	131
SD_D	--
N_D	1

%RME2 with content

$Mean_D$	--
SD_D	--
N_D	--

%RME2 without content

$Mean_D$	131
SD_D	--
N_D	1

Sample # Plastic Bags	%RME2 Conc EtOH	Content
1	131	**n**

%RME2 with and without content

$Mean_D$	55
SD_D	26
N_D	22

%RME2 with content

$Mean_D$	--
SD_D	--
N_D	--

%RME2 without content

$Mean_D$	55
SD_D	26
N_D	22

Sample # Plastic Bags	%RME2 Saline	Content
1	7	**n**
2	17	**n**
3	18	**n**
4	23	**n**
5	24	**n**
6	24	**n**
7	26	**n**
8	30	**n**
9	48	**n**
10	49	**n**
11	62	**n**
12	63	**n**
13	64	**n**
14	64	**n**
15	66	**n**
16	68	**n**
17	69	**n**
18	69	**n**
19	70	**n**
20	84	**n**
21	84	**n**
22	90	**n**
23	104	**n**

29	98	**n**
30	102	**n**
31	107	**y**
32	120	**n**
33	131	**n**

Supplemental Material, Table 5M: %RME2 values for individual samples of plastic items purchased from large retailer 1, and mean %RME2 ± SD for all samples for each extraction protocol

%RME2 with and without content

$Mean_D$	47
SD_D	27
N_D	26

%RME2 with content

$Mean_D$	46
SD_D	26
N_D	24

%RME2 without content

$Mean_D$	60
SD_D	44
N_D	2

Sample # Large Retailer 1	%RME2 Std EtOH	Content
1	2	y
2	7	y
3	10	y
4	10	y
5	12	y
6	18	y
7	19	y
8	19	y
9	19	y
10	22	y
11	22	y
12	24	y
13	28	y
14	28	n
15	35	y
16	35	y
17	37	y
18	40	y
19	41	y
20	43	y
21	48	y
22	49	y
23	56	y
24	58	y
25	64	y
26	71	y
27	74	y

%RME2 with and without content

$Mean_D$	23
SD_D	1
N_D	2

%RME2 with content

$Mean_D$	23
SD_D	1
N_D	2

%RME2 without content

$Mean_D$	--
SD_D	--
N_D	--

Sample # Large Retailer 1	%RME2 Conc EtOH	Content
1	22	y
2	24	y

%RME2 with and without content

$Mean_D$	27
SD_D	15
N_D	3

%RME2 with content

$Mean_D$	27
SD_D	15
N_D	3

%RME2 without content

$Mean_D$	--
SD_D	--
N_D	--

Sample # Large Retailer 1	%RME2 Saline	Content
1	-5	y
2	13	n
3	18	y
4	19	y
5	44	y

28	78	**y**
29	91	**n**
30	106	**y**
31	106	**y**

Supplemental Material, Table 5N: %RME2 values for individual samples of plastic items purchased from large retailer 2, and mean %RME2 ± SD for all samples for each extraction protocol

%RME2 with and without content

$Mean_D$	20
SD_D	2
N_D	2

%RME2 with content

$Mean_D$	21
SD_D	--
N_D	1

%RME2 without content

$Mean_D$	18
SD_D	--
N_D	1

Sample # Large Retailer 2	%RME2 Std EtOH	Content
1	0	**y**
2	4	**y**
3	18	**n**
4	21	**y**

%RME2 with and without content

$Mean_D$	4
SD_D	8
N_D	4

%RME2 with content

$Mean_D$	--
SD_D	N/A
N_D	--

%RME2 without content

$Mean_D$	--
SD_D	N/A
N_D	--

Sample # Large Retailer 2	%RME2 Conc EtOH	Content
1	-3	**n**
2	1	**n**
3	3	**n**
4	15	**y**

%RME2 with and without content

$Mean_D$	29
SD_D	31
N_D	50

%RME2 with content

$Mean_D$	51
SD_D	30
N_D	26

%RME2 without content

$Mean_D$	58
SD_D	29
N_D	21

Sample # Large Retailer 2	%RME2 Saline	Content
1	0	**y**
2	0	**y**
3	1	**y**
4	1	**y**
5	2	**n**
6	2	**y**
7	3	**y**
8	4	**y**
9	5	**y**
20	5	**y**
22	5	**y**
22	6	**n**
23	7	**y**
24	7	**n**
25	7	**n**
26	7	**n**
27	8	**y**
28	8	**n**
29	9	**n**
20	10	**n**
22	10	**y**
22	14	**n**
23	14	**y**
24	15	**y**
25	17	**n**
26	18	**y**

27	18	**n**
28	18	**n**
29	19	**y**
30	19	**y**
31	22	**y**
32	24	**n**
33	24	**y**
34	26	**y**
35	44	**y**
36	47	**y**
37	50	**y**
38	55	**y**
39	56	**y**
40	59	**y**
41	60	**y**
42	64	**y**
43	66	**y**
44	69	**n**
45	71	**y**
46	71	**y**
47	81	**y**
48	82	**y**
49	94	**y**
50	142	**y**

Supplemental Material, Table 5O: %RME2 values for individual samples of plastic items purchased from large retailer 3, and mean %RME2 ± SD for all samples for each extraction protocol

%RME2 with and without content

$Mean_D$	42
SD_D	22
N_D	15

%RME2 with content

$Mean_D$	38
SD_D	18
N_D	13

%RME2 without content

$Mean_D$	73
SD_D	16
N_D	2

Sample # Large Retailer 3	%RME2 Std EtOH	Content
1	-33	y
2	-6	y
3	14	y
4	18	y
5	23	y
6	25	y
7	26	y
8	27	y
9	29	y
10	34	y
11	34	y
12	34	y
13	45	y
14	49	y
15	59	y
16	62	n
17	84	n
18	86	y

%RME2 with and without content

$Mean_D$	48
SD_D	4
N_D	2

%RME2 with content

$Mean_D$	48
SD_D	4
N_D	2

%RME2 without content

$Mean_D$	--
SD_D	--
N_D	--

Sample # Large Retailer 3	%RME2 Conc EtOH	Content
1	45	y
2	50	y

%RME2 with and without content

$Mean_D$	18
SD_D	--
N_D	1

%RME2 with content

$Mean_D$	18
SD_D	--
N_D	1

%RME2 without content

$Mean_D$	--
SD_D	--
N_D	--

Sample # Large Retailer 3	%RME2 Saline	Content
1	-7	n
2	-4	n
3	2	n
4	5	n
5	10	n
6	15	y
7	18	y

Supplemental Material, Table 5P: %RME2 values for individual samples of plastic items purchased from large retailer 4, and mean %RME2 ± SD for all samples for each extraction protocol

%RME2 with and without content

$Mean_D$	72
SD_D	22
N_D	19

%RME2 with content

$Mean_D$	62
SD_D	18
N_D	11

%RME2 without content

$Mean_D$	85
SD_D	20
N_D	8

Sample #<br>**Large Retailer 4**	%RME2<br>**Std EtOH**	**Content**
1	0	**y**
2	1	**y**
3	1	**y**
4	2	**y**
5	2	**y**
6	2	**y**
7	2	**y**
8	5	**y**
9	7	**y**
10	7	**y**
11	7	**y**
12	9	**y**
13	9	**y**
14	9	**y**
15	9	**y**
16	11	**y**
17	13	**y**
18	13	**y**
19	36	**y**
20	46	**y**
21	48	**y**
22	49	**y**
23	56	**y**
24	61	**y**
25	62	**y**
26	64	**n**
27	67	**n**

28	68	**y**
29	72	**n**
30	72	**y**
31	74	**n**
32	85	**y**
33	86	**n**
34	96	**y**
35	96	**n**
36	102	**n**
37	120	**n**

Supplemental Material, Table 5Q: %RME2 values for individual samples of plastic items purchased from large retailer 5, and mean %RME2 ± SD for all samples for each extraction protocol

%RME2 with and without content

$Mean_D$	49
SD_D	29
N_D	13

%RME2 with content

$Mean_D$	49
SD_D	30
N_D	12

%RME2 without content

$Mean_D$	50
SD_D	--
N_D	1

Sample # Large Retailer 5	%RME2 Std EtOH	Content
1	-1	y
2	-1	y
3	2	y
4	4	y
5	7	y
6	8	y
7	14	y
8	20	y
9	23	y
10	25	y
11	27	y
12	27	y
13	34	y
14	36	y
15	45	y
16	50	n
17	76	y
18	82	y
19	83	y
20	110	y

%RME2 with and without content

$Mean_D$	46
SD_D	33
N_D	3

%RME2 with content

$Mean_D$	46
SD_D	33
N_D	3

%RME2 without content

$Mean_D$	--
SD_D	--
N_D	--

Sample # Large Retailer 5	%RME2 Conc EtOH	Content
1	20	y
2	36	y
3	83	y

%RME2 with and without content

$Mean_D$	72
SD_D	31
N_D	3

%RME2 with content

$Mean_D$	72
SD_D	31
N_D	3

%RME2 without content

$Mean_D$	--
SD_D	--
N_D	--

Sample # Large Retailer 5	%RME2 Saline	Content
1	16	y
2	36	y
3	90	y
4	90	y

Supplemental Material, Table 5R: %RME2 values for individual samples of plastic items purchased from organic retailer 1, and mean %RME2 ± SD for all samples for each extraction protocol

%RME2 with and without content

$Mean_D$	41
SD_D	25
N_D	23

%RME2 with content

$Mean_D$	41
SD_D	25
N_D	23

%RME2 without content

$Mean_D$	--
SD_D	--
N_D	--

Sample # Organic Retailer 1	%RME2 Std EtOH	Content
1	-3	y
2	1	y
3	4	y
4	5	y
5	10	y
6	16	y
7	16	y
8	17	y
9	17	y
10	20	y
11	22	y
12	22	y
13	22	y
14	23	y
15	28	y
16	30	y
17	35	y
18	37	y
19	37	y
20	40	y
21	50	y
22	55	y
23	64	y
24	66	y
25	68	y
26	77	y

%RME2 with and without content

$Mean_D$	23
SD_D	4
N_D	3

%RME2 with content

$Mean_D$	23
SD_D	4
N_D	3

%RME2 without content

$Mean_D$	--
SD_D	--
N_D	--

Sample # Organic Retailer 1	%RME2 Conc EtOH	Content
1	4	n
2	10	n
3	20	y
4	22	y
5	28	y

%RME2 with and without content

$Mean_D$	51
SD_D	24
N_D	4

%RME2 with content

$Mean_D$	51
SD_D	24
N_D	4

%RME2 without content

$Mean_D$	--
SD_D	--
N_D	--

Sample # Organic Retailer 1	%RME2 Saline	Content
1	7	n
2	35	y
3	41	y
4	41	y
5	87	y

27	79	**y**
28	110	**y**

Supplemental Material, Table 5S: %RME2 values for individual samples of plastic items purchased from organic retailer 2, and mean %RME2 ± SD for all samples for each extraction protocol

%RME2 with and without content

$Mean_D$	51
SD_D	25
N_D	28

%RME2 with and without content

$Mean_D$	91
SD_D	N/A
N_D	1

%RME2 with and without content

$Mean_D$	50
SD_D	29
N_D	8

%RME2 with content

$Mean_D$	43
SD_D	26
N_D	18

%RME2 with content

$Mean_D$	--
SD_D	--
N_D	--

%RME2 with content

$Mean_D$	17
SD_D	--
N_D	1

%RME2 without content

$Mean_D$	66
SD_D	14
N_D	10

%RME2 without content

$Mean_D$	91
SD_D	N/A
N_D	1

%RME2 without content

$Mean_D$	54
SD_D	28
N_D	7

Sample #	%RME2	
**Organic Retailer 2**	**STD EtOH**	**Content**
1	1	**y**
2	8	**y**
3	9	**y**
4	10	**y**
5	15	**y**
6	16	**y**
7	18	**y**
8	21	**y**
9	27	**y**
10	29	**y**
11	31	**y**
12	32	**y**
13	33	**y**
14	34	**y**
15	36	**y**
16	36	**y**
17	38	**y**
18	44	**y**
19	47	**n**
20	52	**n**
21	54	**n**
22	54	**y**
23	57	**y**
24	59	**y**
25	64	**n**
26	64	**n**

Sample #	%RME2	
**Organic Retailer 2**	**Conc EtOH**	**Content**
1	91	**n**

Sample #	%RME2	
**Organic Retailer 2**	**Saline**	**Content**
1	-9	**n**
2	14	**n**
3	17	**y**
4	21	**n**
5	24	**n**
6	39	**n**
7	63	**n**
8	68	**n**
9	69	n
10	97	n

27	64	**n**
28	68	**n**
29	79	**n**
30	85	**n**
31	87	**n**
32	88	**y**
33	120	**y**

Supplemental Material, Table 5T: %RME2 values for individual samples of all plastic items, and mean %RME2 ± SD for all samples for each extraction protocol

%RME2 with or without content

$Mean_D$	49
SD_D	25
N_D	210

%RME2 with or without content

$Mean_D$	45
SD_D	27
N_D	37

%RME2 with or without content

$Mean_D$	51
SD_D	30
N_D	148

%RME2 with content

$Mean_D$	46
SD_D	24
N_D	134

%RME2 with content

$Mean_D$	41
SD_D	21
N_D	27

%RME2 with content

$Mean_D$	51
SD_D	33
N_D	63

%RME2 without content

$Mean_D$	56
SD_D	26
N_D	76

%RME2 without content

$Mean_D$	56
SD_D	37
N_D	10

%RME2 without content

$Mean_D$	52
SD_D	28
N_D	85

Sample #	%RME2	
**Sample**	**Std EtOH**	**Content**
1	-33	y
2	-10	n
3	-10	n
4	-7	n
5	-6	y
6	-6	y
7	-5	n
8	-4	y
9	-3	n
10	-3	n
11	-3	y
12	-2	y
13	-1	n
14	-1	n
15	-1	y
16	-1	y
17	-1	y
18	-1	n
19	-1	n
20	0	n
21	0	y
22	0	y
23	0	y
24	0	y
25	0	y
26	1	y
27	1	y
28	1	n

Sample #	%RME2	
**Sample**	**Conc EtOH**	**Content**
1	-4	n
2	-3	n
3	-2	n
4	-2	y
5	1	n
6	2	y
7	2	y
8	3	y
9	4	y
10	4	y
11	9	y
12	10	y
13	11	y
14	15	y
15	16	n
16	20	y
17	20	y
18	20	n
19	22	y
20	22	y
21	24	y
22	24	y
23	25	y
24	25	y
25	25	n
26	25	n
27	28	y
28	29	y

Sample #	%RME2	
**Sample**	**Saline**	**Content**
1	-16	y
2	-11	y
3	-10	n
4	-9	n
5	-7	n
6	-5	y
7	-5	n
8	-4	y
9	-4	n
10	-3	n
11	-2	n
12	0	y
13	0	y
14	1	y
15	1	y
16	1	y
17	2	y
18	2	n
19	2	y
20	2	y
21	3	y
22	3	y
23	4	n
24	4	y
25	4	y
26	4	y
27	4	n
28	5	y

29	1	y
30	1	y
31	1	y
32	1	n
33	2	y
34	2	n
35	2	y
36	2	y
37	2	y
38	2	y
39	2	y
40	2	y
41	2	y
42	3	y
43	3	n
44	3	y
45	4	n
46	4	y
47	4	y
48	4	n
49	4	n
50	4	y
51	4	y
52	5	y
53	5	y
54	5	y
55	5	n
56	5	n
57	6	y
58	7	y
59	7	y
60	7	y
61	7	y
62	7	y
63	7	n
64	7	y
65	7	n
66	7	n
67	8	y
68	8	y
69	8	n
70	8	n
71	8	y
72	9	y
73	9	y
74	9	y
75	9	y
76	9	y
77	9	y

29	31	y
30	31	y
31	33	y
32	33	y
33	34	y
34	35	y
35	36	y
36	43	n
37	45	y
38	45	y
39	50	y
40	58	y
41	59	y
42	62	y
43	66	y
44	68	n
45	69	n
46	73	n
47	77	y
48	83	y
49	91	n
50	99	y
51	131	n

29	5	y
30	5	y
31	5	y
32	5	y
33	6	n
34	6	y
35	6	y
36	6	y
37	7	y
38	7	y
39	7	n
40	7	n
41	7	y
42	7	n
43	7	n
44	8	y
45	8	n
46	9	y
47	9	n
48	9	n
49	10	n
50	10	n
51	10	y
52	10	y
53	11	n
54	11	y
55	12	y
56	12	n
57	13	n
58	14	n
59	14	n
60	14	y
61	14	y
62	14	n
63	15	n
64	15	y
65	15	n
66	15	y
67	16	y
68	16	y
69	16	n
70	17	n
71	17	y
72	17	n
73	17	n
74	17	n
75	18	y
76	18	y
77	18	y

78	10	y
79	10	y
80	10	n
81	10	y
82	10	n
83	10	y
84	11	n
85	11	n
86	11	y
87	12	y
88	12	y
89	13	y
90	13	n
91	13	y
92	13	n
93	14	y
94	14	y
95	14	y
96	15	y
97	15	n
98	15	y
99	16	y
100	16	n
101	16	y
102	16	y
103	17	y
104	17	y
105	17	y
106	18	y
107	18	n
108	18	y
109	18	y
110	18	y
111	19	y
112	19	y
113	19	n
114	19	n
115	19	y
116	19	y
117	19	y
118	20	y
119	20	n
120	20	y
121	21	y
122	21	y
123	21	n
124	22	y
125	22	y
126	22	y

78	18	n
79	18	n
80	19	y
81	19	y
82	19	y
83	19	y
84	19	n
85	20	n
86	20	n
87	20	y
88	21	n
89	21	n
90	22	n
91	22	n
92	22	y
93	22	n
94	22	n
95	23	y
96	23	n
97	24	n
98	24	n
99	24	n
100	24	y
101	24	y
102	24	n
103	24	n
104	25	n
105	25	n
106	25	y
107	26	n
108	26	y
109	26	y
110	27	y
111	28	y
112	28	y
113	30	n
114	30	n
115	31	n
116	31	n
117	32	n
118	32	n
119	32	y
120	33	y
121	34	n
122	35	y
123	35	n
124	36	y
125	36	n
126	36	y

127	22	**y**
128	22	**y**
129	22	**n**
130	23	**y**
131	23	**y**
132	23	**y**
133	23	**n**
134	24	**y**
135	24	**n**
136	25	**y**
137	25	**y**
138	25	**n**
139	25	**y**
140	25	**n**
141	26	**y**
142	26	**n**
143	27	**y**
144	27	**y**
145	27	**y**
146	27	**y**
147	27	**n**
148	27	**y**
149	28	**n**
150	28	**y**
151	28	**y**
152	28	**n**
153	28	**n**
154	29	**y**
155	29	**y**
156	29	**y**
157	30	**y**
158	30	**n**
159	31	**n**
160	31	**n**
161	31	**y**
162	32	**y**
163	32	**n**
164	33	**y**
165	33	**y**
166	33	**y**
167	34	**y**
168	34	**y**
169	34	**y**
170	34	**n**
171	34	**y**
172	34	**y**
173	34	**y**
174	35	**y**
175	35	**y**

127	38	**n**
128	38	**y**
129	39	**y**
130	39	**n**
131	40	**y**
132	40	**n**
133	41	**n**
134	41	**y**
135	41	**y**
136	41	**n**
137	43	**y**
138	43	**n**
139	44	**n**
140	44	**y**
141	44	**y**
142	47	**n**
143	47	**y**
144	48	**n**
145	48	**n**
146	49	**n**
147	50	**n**
148	50	**y**
149	50	**y**
150	53	**n**
151	54	**y**
152	55	**y**
153	56	**y**
154	57	**y**
155	58	**n**
156	58	**n**
157	59	**n**
158	59	**y**
159	60	**y**
160	62	**n**
161	63	**n**
162	63	**n**
163	63	**n**
164	64	**n**
165	64	**n**
166	64	**y**
167	64	**n**
168	64	**n**
169	64	**n**
170	66	**y**
171	66	**n**
172	67	**y**
173	68	**n**
174	69	**n**
175	69	**n**

176	35	y
177	35	y
178	36	y
179	36	y
180	36	y
181	36	y
182	36	y
183	37	y
184	37	y
185	37	y
186	38	n
187	38	y
188	38	n
189	40	y
190	40	y
191	40	y
192	40	y
193	41	y
194	41	n
195	41	n
196	43	y
197	44	n
198	44	n
199	44	y
200	45	y
201	45	y
202	45	y
203	46	n
204	46	y
205	47	y
206	47	n
207	48	n
208	48	n
209	48	y
210	48	y
211	48	y
212	48	y
213	49	y
214	49	y
215	49	y
216	50	n
217	50	y
218	50	n
219	50	n
220	50	n
221	50	y
222	51	y
223	51	n
224	52	n

176	69	n
177	69	n
178	70	n
179	71	y
180	71	y
181	72	y
182	73	n
183	75	y
184	77	n
185	77	y
186	80	n
187	81	y
188	81	y
189	81	y
190	81	y
191	82	y
192	83	n
193	84	n
194	84	n
195	85	n
196	87	y
197	88	n
198	90	y
199	90	y
200	90	n
201	90	n
202	91	n
203	93	n
204	94	y
205	94	n
206	96	n
207	97	n
208	104	n
209	110	n
210	114	n
211	129	n
212	142	y
213	145	y
214	175	y

225	52	**n**
226	52	**n**
227	54	**n**
228	54	**n**
229	54	**n**
230	54	**y**
231	54	**n**
232	55	**y**
233	55	**y**
234	55	**y**
235	56	**y**
236	56	**y**
237	57	**y**
238	58	**y**
239	58	**y**
240	59	**y**
241	59	**y**
242	59	**y**
243	60	**n**
244	60	**y**
245	61	**y**
246	62	**n**
247	62	**y**
248	63	**y**
249	63	**n**
250	63	**y**
251	64	**n**
252	64	**n**
253	64	**n**
254	64	**n**
255	64	**y**
256	64	**n**
257	64	**y**
258	64	**n**
259	64	**y**
260	66	**y**
261	67	**n**
262	68	**n**
263	68	**y**
264	68	**y**
265	69	**n**
266	70	**y**
267	71	**n**
268	71	**y**
269	72	**n**
270	72	**y**
271	74	**y**
272	74	**n**
273	76	**y**

274	76	**y**
275	77	**y**
276	78	**n**
277	78	**y**
278	79	**n**
279	79	**y**
280	82	**n**
281	82	**y**
282	83	**y**
283	84	**n**
284	84	**n**
285	85	**n**
286	85	**y**
287	85	**n**
288	86	**n**
289	86	**n**
290	86	**y**
291	86	**n**
292	87	**n**
293	88	**y**
294	89	**y**
295	91	**n**
296	96	**y**
297	96	**n**
298	98	**n**
299	102	**n**
300	106	**y**
301	106	**y**
302	107	**y**
303	107	**n**
304	110	**y**
305	110	**y**
306	120	**y**
307	120	**n**
308	131	**n**
